# Supplementary figures and images for: Isthminia panamensis, a new fossil inioid (Mammalia, Cetacea) from the Chagres Formation of Panama and the evolution of ‘river dolphins’ in the Americas
Source: PeerJ. 2015 Sep 1;3:e1227. doi: 10.7717/peerj.1227 (PMC4562255; doi:10.7717/peerj.1227)

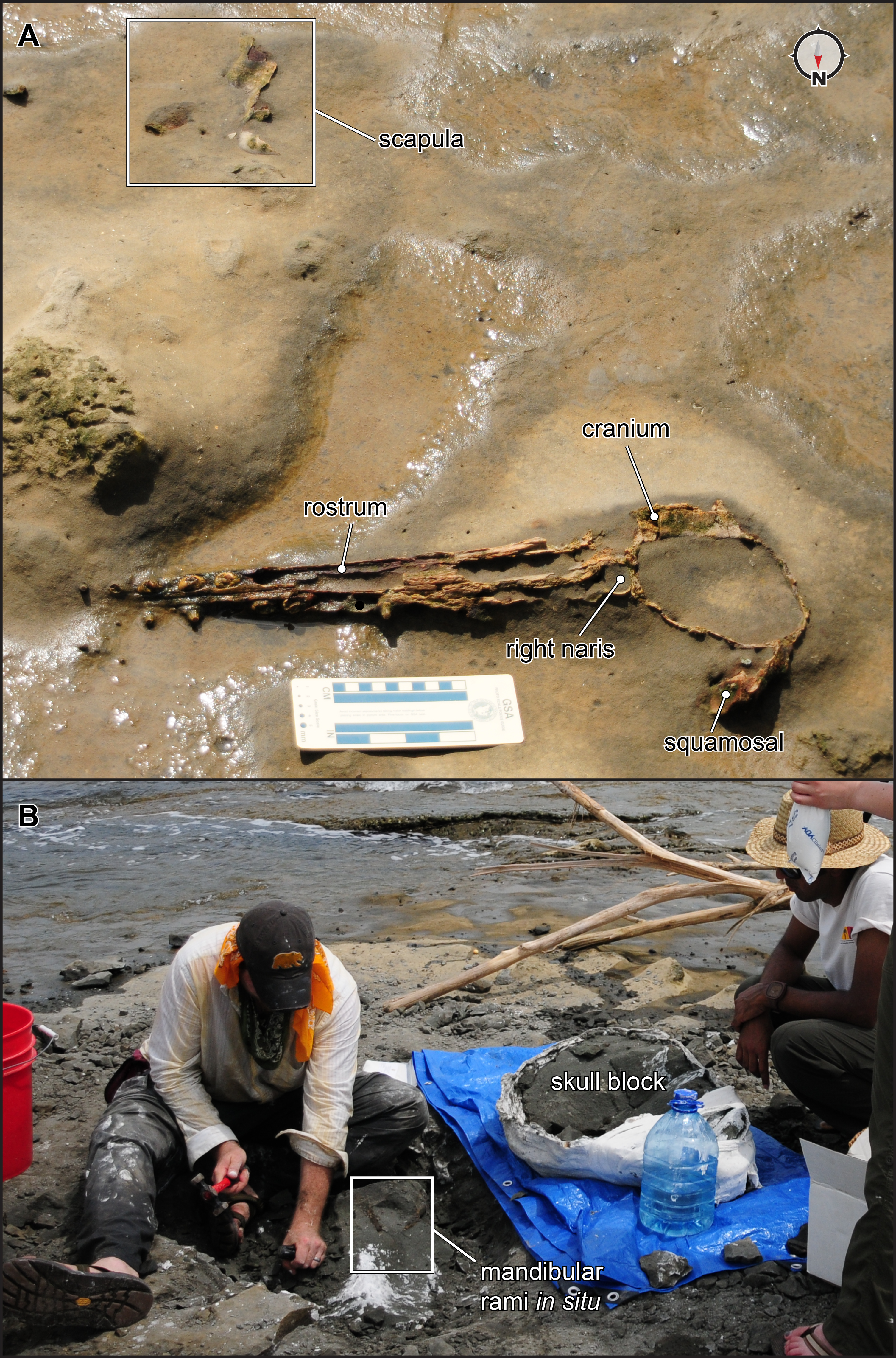

Supplement: Figure S1 — (A) The specimen exposed in the outcrop, at low tide, with the scapula, oriented lateral side facing stratigraphic up, approximately 35 cm away from the skull, which was exposed ventral side up. Scale bar = 10 cm. Photo: J. Velez-Juarbe. (B) With the high tide returning, removal of the plaster jacketed sediment block, containing the skull, exposed the mandibles located directly underneath it. The mandible was oriented dorsal surface facing stratigraphic up. Photo: A. O’Dea. [file peerj-03-1227-s001.jpg]
