# Supplementary material for: Isthminia panamensis, a new fossil inioid (Mammalia, Cetacea) from the Chagres Formation of Panama and the evolution of ‘river dolphins’ in the Americas
Source: PeerJ. 2015 Sep 1;3:e1227. doi: 10.7717/peerj.1227 (PMC4562255; doi:10.7717/peerj.1227)
Supplement: File S1 [file peerj-03-1227-s002.docx]

Supplemental File S1

***Isthminia panamensis*, a new fossil inioid (Mammalia: Cetacea) from the Chagres Formation of Panama and the evolution of ‘river dolphins’ in the Americas**

Character matrix for this study, modified from Aguirre-Fernández & Fordyce (2014), with coding for *Isthminia* *panamensis* and an updated the character scoring for *Ischyrorhynchus*, and references.

Table of Content:

1. Character-State List p. S2

2. Matrix p. S41

3. References p. S93

**1. List of Characters and States**

The character state list used in our analysis follows that of Aguirre and Fordyce (2014) which stems from the list used by Geisler et al. (2012) which focused on Inioidea.

1. *Baleen*: (0) absent; (1) present.

2. *Rostrum*: (0) narrows in width anteriorly or anterior half approximately the same width as posterior half; (1) anterior part widened transversely.

3. *Length of rostral portion of maxilla*: (0) short, rostral portion of maxilla < 43% of condylobasal length excluding the premaxillae; (1) intermediate, rostral portion between 48 and 70% of modified condylobasal length; (2) elongate, rostral portion >73% modified condylobasal length.

4. *Anterior half of maxilla*: (0) its lateral edge in cross section forms an angle of 60º to 45º; (1) highly acute angle with flattened maxilla.

5. *Vomerine trough, or mesorostral canal*: (0) open, vomer in cross section is V-shaped or U-shaped; (1) partially or completely filled with bone, becomes solid rod of bone.

6. *Rostral constriction well anterior to antorbital notch*: (0) absent; (1) present.

7. *Width of rostrum at antorbital notch*: (0) wide, rostral width >92% the width across of middle of orbits; (1) fairly wide, between 82% and 72% the width across orbits; (2) narrow, between 68% and 46% the orbital width; (3) very narrow, between 32% and 29% the orbital width.

8. *Premaxilla in dorsal view*: (0) Portion adjacent to and anterior to nasal opening narrows or remains the same width anteriorly; (1) widens at anterior end.

9. *Premaxillae on anterior two thirds of rostrum*: (0) with skull in dorsal view, contact along midline for most or entire length; (1) sporadic contact along rostrum; (2) separated by a narrow fissure for entire length; (3) clear separation although mesorostral gutter still has a partial roof; (4) very wide separation, mesorostral gutter is completely open along entire length.

10. *Suture between maxilla and premaxilla on rostrum*: (0) suture fused along most of rostrum; (1) anterior quarter of rostrum fused with remaining portions unfused; (2) unfused long entire rostrum but articulation tight; (3) suture is unfused and marked by a deep groove.

11. *Posterior region of rostral edge*: (0) lateral margin is straight or gently concave with skull in dorsal view; (1) slightly bowed outward causing a V-shaped antorbital notch; (2) bowed far outward forming a deep U-shaped antorbital notch; (3) lateral margin of maxilla nearly contact lacrimal and jugal resulting in the opening of the notch being a narrow slit.

12. *Posterodorsal portion of maxilla*: (0) sutured to frontal; (1); not sutured, separated from frontal by distinct gap, which is situated between the maxilla anterodorsal and the frontal ventrally

13. *Steep face on anterolateral edge of zygmatic process of maxilla clearly separating it from rostral portion of maxilla*: (0) absent; (1) present but low; (2) present and well developed.

14. *Posterior end of ascending process of maxilla*: (0) tapers to a point; (1) end is squared-off.

15. *Posterior wall of antorbital notch*: (0) maxilla; (1) lacrimal and jugal.

16. *Palatal surface of rostrum*: (0) flat or gently concave; (1) bears pronounced longitudinal keel along the midline of the rostrum.

17. *Palatal surface of maxilla*: (0) bears few vascular foramina, those that are present are small; (1) bears many, large vascular foramina that open laterally and anterolaterally into long sulci; (2) bears numerous small vascular foramina that lack sulci.

18. *Posterior end of palatal surface of rostrum at the suture between palatine and maxilla*: (0) concave to flat, depth of rostrum, measured as the dorsoventral distance from the level of the lateral edge of rostrum to the ventral-most part of rostrum, is <8% the width of rostrum at antorbital notches; (1) convex, depth between 11% and 25% the rostral width (2) highly convex, rostral depth >27% the rostral width.

19. *Palatine*: (0) sutured to maxilla and suture visible; (1) fused to maxilla.

20. *Palatine/Maxilla suture*: (0) in ventral view, suture between both palatine and both maxillae is straight transversely or bowed anteriorly; (1) maxillae have posterior processes that separate palatines anteriorly, suture around midline is V-shaped and points posteriorly.

21. *Teeth in females*: (0) erupt in adulthood; (1) do not erupt in adulthood but remain in the crypt.

22. *Tooth rows*: (0) separated and diverge posteriorly; (1) left and right sides adjacent to the midline and thus close together, are nearly parallel.

23. *Number of double-rooted teeth in maxilla*: (0) none; (1) 1 or 2; (2) 4; (3) 5; (4) 6; (5) 7; (6) 8 or more.

24. *Number of teeth with alveoli completely enclosed in the maxilla*: (0) none, 1, 2, or 3; (1) 7 to 8; (2) 9 to 10; (3) 11 to 13; (4) 15 to 17; (5) 21 to 23; (6) 26 to 29; (7) 32 to 39; (8) 50 to 60.

25. *Large diastemata between posterior buccal teeth*: (0) absent; (1) present.

26. *Tooth enamel*: (0) smooth; (1) bear reticulating striae; (2) nodular.

27. *Lower anterior mandibular teeth*: (0) conical; (1) spatulate; (2) laterally compressed

28. *Lower anterior teeth*: (0) deeply rooted with at least half of tooth forming root; (1) not deeply rooted.

29. *Accessory shelf on posterior teeth*: (0) present; (1) absent.

30. *Posterior buccal teeth*: (0) high peg-shaped teeth, crown base is <37% the crown height; (1) nearly an equilateral triangle, crown base is between 100% to 148% the crown height; (2) wide low teeth, crown base is >180% the crown height.

31. *Buccal teeth ectocingulum*: (0) absent; (1) present.

32. *Buccal teeth entocingulum*: (0) present; (1) absent.

33. *Buccal teeth*: (0) bear accessory cusps; (1) cusps absent.

34. *Central cusp as compared to denticles*: (0) much larger; (1) subequal.

35. *Anterior-most mandibular teeth*: (0) oriented anteriorly; (1) vertical; (2) inclined posteriorly.

36. *Anterior-most mandibular teeth*: (0) smaller than posterior teeth; (1) approximately same size as posterior teeth; (2) greatly enlarged.

37. *Number of teeth in lower jaw*: (0) none; (1) one; (2) two; (3) 8 to 9; (4) 11 to 12; (5) 13 to 14; (6) 20 to 23; (7) 24 to 27; (8) 28 to 34; (9) more than 40 teeth.

38. *Mandible in lateral view*: (0) straight; (1) arched dorsally.

39. *Length of mandibular symphysis*: (0) short, mandibular symphysis forms less than 28% of the total mandibular length; (1) long, symphysis length between 33% and 40% of the mandibular length; (2) very long, symphysis forms more than 48% of the length of the mandibles.

40. *Mandibular symphysis*: (0) fused; (1) sutured but unfused; (2) not sutured, connected by ligaments.

41. *Longitudinal groove on underside of mandible*: (0) absent; (1) present.

42. *Mandible*: (0) bowed medially; (1) straight; (2) slightly bowed laterally, a line drawn from the posterior-most to anterior-most points stays within body of mandible; (3) strongly bowed outward, line from anterior to posterior point does not entirely lie within body of mandible.

43. *Mandibular fossa*: (0) small or absent; (1) present and large, forms a large cavity posterior to mandibular foramen.

44. *Shape of coronoid process*: (0) long and low, height of mandible at coronoid process <89% the length of coronoid; (1) height of mandible between 100% and 177% the length of the coronoid; (2) short and high, height between 203% and 300% coronoid length; (3) very hight and short, height >450% the length of the coronoid.

45. *Dorsal surface of condyle*: (0) elevated above dorsal edge of the rest of the mandible, not counting coronoid process; (1) at same level as rest of mandible.

46. *Supraorbital processes of frontal*: (0) are horizontal or gradually slope lateroventrally away from vertex of skull; (1) abruptly depressed at base to a level noticeably below that of dorsal surface of interorbital region; (2) slope laterodorsally away from vertex.

47. *Dorsal edge of orbit relative to lateral edge of rostrum*: (0) below the level of the edge of the rostrum; (1) orbit low, either in line with edge of rostrum or slightly above it, height of orbit <46% the height of rostral base, both height measured relative to the lateral edge of rostrum; (2) orbit low, height of dorsal edge of orbit between 50% and 92% the rostral height; (3) orbit high, height between 100% and 128% the rostral height; (4) orbit elevated well above rostrum, orbital height >163% the rostral height.

48. *Frontal/Maxilla suture*: (0) with skull in lateral view, suture is approximately horizontal, and lateral exposure of frontal over the orbit does not thicken posteriorly; (1) angled posterodorsally at an angle of 50% to 70% from axis of rostrum, lateral exposure of frontal thickens posteriorly.

49. *Anterior edge of the supraorbital process*: (0) oriented anteromedially; (1) oriented slightly anterolaterally, forms an angle <30º with sagittal plane; (2) oriented anterolaterally, forms an angle between 35º and 60º; (3) oriented anterolaterally or laterally, forms an angle between 68º and 90º; (4) oriented posterolaterally, forms an angle between 107º and 120º; (5) oriented posterolaterally, forms an angle >142º.

50. *Lacrimal*: (0) forms small bone on anterior edge of orbit with small orbital portion; (1) enlarged both posteromedially and anterolaterally paralleling anterior edge of supraorbital process of frontal, shaped like a thick rod.

51. *Lacrimal*: (1) restricted below supraorbital process of frontal; (1) wraps around anterior edge of supraorbital process of frontal and slightly overlies its anterior end; (2) greatly expanded posterodorsally and covering much of lateral side of supraorbital process of frontal.

52. *Lacrimal foramen or groove*: (0) present; (1) absent.

53. *Lacrimal and jugal*: (0) separate; (1) fused.

54. *Jugal and lacrimal*: (0) jugal and lacrimal contact each other externally; (1) lacrimal excluded from edge of skull, jugal directly contacts anterior edge of frontal.

55. *Combined anteroposterior length of the lacrimal and jugal exposure posterior to antorbital notch*: (0) with skull in ventral view, exposure is small and combined length forms <31% of anteroposterior distance from antorbital notch to postorbital ridge; (1) intermediate, forms between 50% and 92% of that distance; (2) large, forms between 62% and 69% that distance; (3) very large, forms >77% of that distance.

56. *Jugal*: (0) thick and sturdy; (1) thin splint or incomplete or absent.

57. *Dorsolateral edge of internal opening of infraorbital foramen*: (0) formed by maxilla; (1) formed by maxilla and lacrimal and/or jugal; (2) formed by lacrimal and/or jugal; (3) formed by frontal.

58. *Ventromedial edge of internal opening of infraorbital foramen*: (0) formed by maxilla; (1) formed by maxilla and palatine and/or pterygoid; (2) formed by palatine and/or pterygoid.

59. *Maxillary infraorbital plate*: (0) absent; (1) present but small; (2) present and large.

60. *Anterior-most point on the posterior edge of the supraorbital process*: (0) the anterior-most point is at the lateral edge of postorbital process; (1) located laterally, between 70% and 74% of transverse distance from sagittal plane to the lateral edge of postorbital process; (2) positioned approximately midway, located between 42% and 61% of that distance; (3) medially positioned, located at a point <34% of that distance.

61. *Postorbital process*: (0) long and projects posterolaterally and slightly ventrally; (1) short and directed ventrally.

62. *Postorbital ridge*: (0) present, forms well-defined curved ridge on posterior edge of sulcus for optic nerve; (1) no well-defined ridge, region is gently convex.

63. *Facial region of skull, skull in lateral view*: (0) concave; (1) flat; (2) moderately arched dorsally; (3) greatly arched dorsally.

64. *Infraorbital foramina*: (0) single; (1) two; (2) three or more.

65. *Rostral basin*: (0) absent or poorly defined; (1) present, situated medial to antorbital notch and anterior to supraorbital process of frontal, best developed medially and ventrally where lateral edge of maxilla is very thin.

66. *Transverse distance between lateral edges of right and left premaxillae at antorbital notches*: (0) small, distance <48% the width of rostrum at antorbital notches; (1) intermediate, distance between 52% and 64% the antorbital width; (2) wide, distance > than 78% the antorbital width.

67. *Premaxillae immediately anterior to external bony nares*: (0) widely separate with skull in dorsal view, gap between medial edges of premaxillae >63% the maximum width of external bony nares; (1) narrow separation, gap between premaxillae between 56% and 32% the width of external nares; (2) separation absent or nearly so, gap < 28% the nares width.

68. *Premaxillae anterior to nasal openings*: (0) are flat or concave, form a premaxillary sac fossa (spiracular plate); (1) convex transversely; (2) form distinct bosses or "premaxillary eminencies" with steep posterior faces on anterior edges of nasal openings.

69. *Premaxillary foramina*: (0) absent; (1) present and one on right side; (2) two on right side; (3) three on right side.

70. *Premaxillary foramen size*: (0) right and left subequal; (1) left larger than right; (2) left much larger than right.

71. *Position of premaxillary foramen*: (0) far anterior to antorbital notch and anterior edge of supraorbital process; (1) approximately medial to or posterior to antorbital notch region, which is at the junction of supraorbital process with rostrum.

72. *Posterolateral sulcus from premaxillary foramen*: (0) sulcus very short or absent; (1) present and short; (2) present and extends to level equivalent to middle of nasal openings.

73. *Premaxillae*: (0) restricted to medial position adjacent to mesorostral canal and nasal opening; (1) extended laterally covering much of the supraorbital process.

74. *Posterior-most end of ascending process of premaxilla*: (0) located just anterior to or in a transverse line with anterior edge of supraorbital process of the frontal; (1) in line with anterior half of supraorbital process of frontal or halfway point, anteroposteriorly of supraorbital process; (2) in line with posterior half of supraorbital process or postorbital process of frontal; (3) in line with gap between postorbital process and anterior tip of zygomatic process of the squamosal or in line with anterior tip of the latter process; (4) in line with space between anterior tip of zygomatic process of squamosal and anterior edge of floor of the squamosal fossa or in line with anterior edge of floor of the squamosal fossa; (5) located posterior to anterior edge of floor of the squamosal fossa.

75. *Maxillary foramen*: (0) absent; (1) present and one, situated over supraorbital process of frontal; (2) two; (3) foramina absent because roof of canal that carries posterior branches of internal maxillary artery and the maxillary division of infraorbital nerve is unossified.

76. *Maxilla*: (0) abuts anterior edge of supraorbital process of frontal; (1) partially covers supraorbital process; (2) covers almost entire surface.

77. *Posterior-most edge of the ascending process of maxilla*: (0) situated well anterior to anterior edge of orbit; (1) in transverse line with anterior half of supraorbital process of frontal or in line with the halfway point, anteroposteriorly, of supraorbital process; (2) in line with posterior half of supraorbital process or in line with postorbital process of frontal; (3) in line with gap between postorbital process and the anterior tip of zygomatic process of squamosal or in line with anterior tip of the latter process; (4) in line with space between anterior tip of zygomatic process of squamosal and anterior edge of floor of squamosal fossa or in line with anterior edge of the floor of squamosal fossa; (5) posterior to anterior edge of floor of squamosal fossa.

78. *Anterolateral corner of maxilla overlying supraorbital process of frontal*: (0) thin and equal in thickness to parts posteromedial; (1) thickened with thinner maxilla in posteromedial direction.

79. *Maxillary ridge*: (0) absent; (1) present; (2) form transversely compressed and high crest; (3) crest arches over and encloses a cavity for the melon.

80. *Anterior edge of nasals*: (0) in transverse line with incisors, canines, or intervening diastema; (1) in line with P1; (2) in line with P2 or about 18% of the total rostral length towards anterior edge of rostrum; (3) just anterior to or in line with anterior edge of supraorbital process of frontal; (4) in line with anterior half of supraorbital process of frontal or in line with the halfway point, anteroposteriorly, of supraorbital process; (5) in line with posterior half of supraorbital process or in line with postorbital process of frontal; (6) in line with gap between postorbital process and the anterior tip of zygomatic process of squamosal or in line with the anterior tip of the latter process; (7) in line with space between the anterior tip of zygomatic process of squamosal and anterior edge of the floor of squamosal fossa or in line with anterior edge of the floor of squamosal fossa; (8) posterior to the anterior edge of the floor of the squamosal fossa.

81. *Anterior edge of nasal openings*: (0) V-shaped, premaxillae gradually converge anteriorly to the midline; (1) U-shaped, premaxillae abruptly converge anteriorly to the midline.

82. *Maxillae*: (0) in region anterior to nasal openings, maxillae are exposed lateral to premaxillae; (1) maxillae are exposed at posterior end of roof of mesorostral gutter, medial to the premaxillae and nearly converge on midline; (2) same as 1 except maxilla also exposed on anterior edge of nasal openings.

83. *Ossicles*: (0) absent; (1) present, occur in anteromedial corners of nasal openings, probably a derivative of the maxilla.

84. *Right premaxilla*: (0) posterior edge approximately in line with posterior edge of left premaxilla; (1) right premaxilla extended distinctly farther than left; (2) right extended much farther than left.

85. *Transverse width of right premaxilla immediately anterior to external bony nares*: (0) distinctly narrower than left premaxilla; (1) subequal, width of right premaxilla within 10% of the width of left premaxilla; (2) right wider, width is between 130% and 145% the width of the left; (3) right much wider, width > 167% the width of the left.

86. *Right premaxilla*: (0) portion posterior to nasal openings wider than portion anterior to opening, with nasal septum angled anteriorly and to the right; (1) portion anterior wider than portion posterior to nasal opening, septum angled anteriorly and to the left.

87. *Osseous external nasal openings*: (0) left and right are the same size; (1) left is twice or more the size of the right.

88. *Supracranial basin*: (0) absent; (1) present.

89. *Posterior end of premaxilla*: (0) Posterior end adjacent to lateral edge of nasal opening; (1) angled slightly laterally resulting in the following sequence, from lateral to medial, in one transverse plane: premaxilla, maxilla, anterior edge of nasals or mesethmoid.

90. *Angle of premaxillae anterior to external bony nares, skull in lateral view*: (0) low angle, premaxillae form an angle < 28° with the lateral edge of rostrum; (1) intermediate angle, form an angle between 30° and 40°; (2) high angle, form an angle > 45°.

91. *Premaxillae adjacent to nasal opening*: (0) thin dorsoventrally and porous internally; (1) pachyostotic, in direction perpendicular to face, and pachyosteosclerotic but nasals and premaxillae equally project dorsally and anteriorly; (2) extreme pachyostosis, premaxillae adjacent to nasals project farther outward, anteriorly and dorsally.

92. *Proximal ethmoid region*: (0) not visible in dorsal view, roofed over by nasals; (1) exposed dorsally.

93. *Mesethmoid*: (0) forms T-shaped bone with median plate separating right and left nasal passages, not all of the dorsal part is divided by median plate; (1) bears expanded posterodorsal plate which is not divided by median plate, median plate situated more ventrally.

94. *Shape of soft tissue external nares*: (0) crescent with apices pointed anteriorly; (1) crescent with apices pointed posteriorly, might be skewed; (2) rectangular; (3) a longitudinal slit, might be slightly sigmoidal or angled; (4) comma-shaped.

95. *Soft tissue nasal passages distal to bony external nares*: (0) separate with two separate soft tissue external nares; (1) separate for most of length but confluent just proximal to blowhole; (2) confluent.

96. *Orientation of right soft tissue nasal passages*: (0) oriented anterodorsally; (1) oriented dorsally.

97. *Right posterior dorsal bursa*: (0) small; (1) hypertrophied to form the spermaceti organ.

98. *Melon*: (0) absent; (1) small; (2) hypertrophied.

99. *Distal sacs*: (0) absent; (1) present, situated immediately distal to museau de singe.

100. *Left and right distal sacs*: (0) equal in size; (1) right larger, includes physeterid condition of no distal sac on the left nasal passage.

101. *Blowhole ligament*: (0) absent; (1) present.

102. *Right posterior nasal sac*: (0) present and elongate, in some cases it reaches osteological vertex; (1) reduced and short, never reaches osteological vertex; (2) absent.

103. *Nasofrontal sacs*: (0) absent; (1) portions posterior to nasal passage present.

104. *Inferior vestibule*: (0) absent; (1) present, forms a diverticulum of nasal passages posterior to blowhole ligament.

105. *Premaxillary sacs*: (0) absent; (1) present, form the most proximal diverticulum of nasal passages; they extend anteriorly adjacent to dorsal surfaces of premaxillae.

106. *Accessory sac*: (0) absent; (1) present, forms a small diverticulum of inferior vestibule and extends anterolaterally around the attachment of blowhole ligament to premaxilla.

107. *Inflection of ascending process of premaxilla*: (0) gradual with premaxilla in dorsal view smoothly tapering as premaxilla shifts from a horizontal to a mostly vertical position; (1) abrupt with an anterior splint of maxilla that emarginates the posterior edge of premaxilla and splits it into a posterolateral plate and posteromedial splint.

108. *Position of inflection of premaxilla*: (0) in transverse line with P1; (1) in line with P2 or about 18% of total rostral length towards anterior edge of rostrum; (2) just anterior to or in line with anterior edge of supraorbital process of frontal; (3) in line with anterior half of supraorbital process of frontal or in line with the halfway point, anteroposteriorly, of supraorbital process; (4) in line with posterior half of supraorbital process or in line with postorbital process of frontal; (5) in line with gap between postorbital process and the anterior tip of zygomatic process of squamosal or in line with the anterior tip of the latter process, space is absent in some taxa; (6) in line with space between the anterior tip of zygomatic process of squamosal and anterior edge of the floor of squamosal fossa or in line with anterior edge of the floor of squamosal fossa; (7) posterior to anterior edge of the floor of squamosal fossa.

109. *Premaxillary cleft*: (0) absent; (1) present, posterior part of ascending process of premaxilla bears a distinct cleft that originates from at posterior edge of the premaxilla and continues anteriorly dividing the premaxilla in two; (2) present but cleft is shallow.

110. *Premaxillae adjacent to and at posterior edge of the nasal opening*: (0) do not clearly overhang maxillae; (1) premaxillae overhang maxillae; (2) premaxillae greatly enlarged laterally, region between lateral edge of the right premaxilla and supraoccipital is partially enclosed.

111. *Narial pit*: (0) absent; (1) present, an anterior extension of nasal passage and forms a blind pocket in the maxilla just dorsal to maxilla/palatine suture; medial wall of pocket is formed by vomer.

112. *Posterior end of ascending process of premaxillae*: (0) face anterolaterally; (1) face anteriorly; (2) face anteromedially.

113. *Nasal bones*: (0) two; (1) one; (2) none.

114. *Suture between right and left nasals and right and left frontals*; (0) shifted towards right side; (1) situated on midline; (2) shifted towards left side.

115. *Dorsoventral thickness of anterior edge of nasal*: (0) very thin, nasal thickness < 82% of the anterior nasal width; (1) thick, nasal thickness between 100% and 173% of the nasal width; (2) very thick, > 200% the nasal width.

116. *Both nasals in dorsal view*: (0) anterior edges are straight in one transverse plane; (1) with point on midline and a gap on each side between premaxilla and nasal.

117. *Nasal*: (0) elongate anteroposterior plate or blocky; (1) anteroposteriorly compressed into a nearly vertical plate with fossa on ventrolateral surface for posterior nasal sac; (2) fossa well excavated with boss delimiting dorsal edge.

118. *Nasals*: (0) medial portions roughly in same horizontal plane as lateral portions or higher; (1) medial portions greatly depressed forming a median trough immediately posterior to nasal openings.

119. *Maximum transverse width of both nasals*: (0) very narrow, < 37% of the maximum width of the external bony nares; (1) narrow, width between 55% and 89% of the nares width; (2) within 10% of the nares width; (3) wide, width between 123% and 140% the nares width; (4) very wide, between 152% and 160% the nares width; (5) extremely wide, width > 188% the width of external bony nares.

120. *Combined width of posterior edge of nasals*: (0) wide, width > 150% the maximum width of external bony nares; (1) subequal to external nares, width between 85% and 135% the nares width; (2) narrow, width between 79% and 50% the nares width; (3) very narrow, width between 44% and 39% the nares width; (4) extremely narrow, width < 31% the nares width.

121. *Nasal/Frontal suture*: (0) approximately straight transversely; (1) frontal has anterior wedge between posterior ends of nasals.

122. *Position of posterior-most edge of nasals*: (0) just anterior to or in a transverse line with anterior edge of supraorbital process of frontal; (1) in line with anterior half of supraorbital process of frontal or in line with the halfway point, anteroposteriorly, of supraorbital process; (2) in line with posterior half of supraorbital process or in line with postorbital process of frontal; (3) in line with gap between postorbital process and the anterior tip of zygomatic process of squamosal or in line with the anterior tip of the latter process; (4) between anterior the tip of zygomatic process of squamosal and anterior edge of the floor of squamosal fossa or in line with anterior edge of the floor of squamosal fossa; (5) posterior to the anterior edge of the floor of the squamosal fossa.

123. *Height of posterior portions of nasals relative to lateral edge of maxilla*: (0) approximately equal to height of the base of rostrum (see character 58 for a description of measurement used); nasal height between 92% and 139% the rostral height; (1) elevated above rostrum, height of nasals between 156% and 203% the rostral height; (2) very elevated, height of nasals between 229% and 282% the rostral height; (3) extremely elevated, height of nasals between 354% and 420% the rostral height; (4) nasals tower above facial part of skull, height of nasals > 548% the rostral height.

124. *Frontals*: (0) lower than nasals; (1) same height as nasals; (2) higher than nasals.

125. *Frontals posterior to nasals and between the premaxillae*: (0) wider than maximum transverse width across nasals; (1) same width as nasals; (2) narrower than nasals, maxillae expanded medially posterior to the nasals.

126. *Dorsal exposure of frontals*: (0) fairly flat with separation between right and left frontal obscure; (1) frontals are nodular with distinct separating sulcus on midline.

127. *Anterodorsal wall of braincase*: (0) formed by frontal; (1) mostly formed by maxilla.

128. *Supraoccipital*: (0) below frontals and/or nasals, whichever is higher; (1) at same level as frontals and/or nasals; (2) higher than frontals and/or nasals.

129. *Maxilla on dorsal surface of skull*: (0) does not contact supraoccipital posteriorly, maxilla separated by frontal and/or parietal; (1) contact present.

130. *Temporal crest*: (0) dorsal surface adjacent to crest is nearly horizontal, crest appears to be directed laterally; (1) dorsal surface is concave and surface of temporal fossa below crest faces almost entirely laterally, crest appears to be oriented dorsolaterally.

131. *Temporal crest*: (0) in posterior position, frontal roofs over the anterior third or more of temporal fossa; (1) on posterior edge of supraorbital process of frontal; (2) lateral end of temporal crest on dorsal surface of supraorbital process; (3) entire crest on dorsal surface of supraorbital process.

132. *Roof of temporal fossa*: (0) frontal; (1) frontal but with large opening through which maxilla and/or premaxilla is exposed; margins of the window are formed by a frontal ring.

133. *Frontal/parietal suture in lateral view*: (0) vertical or slightly angled posteroventrally; (1) dorsal portion of suture pointed and extended far anterior so that the anteriormost point of parietal is anterior to the posterior-most point of premaxilla.

134. *Parietals in dorsal view*: (0) contact each other on the midline or are separated by an interparietal; (1) are in place in the skull roof but are visible only as small triangular areas at edges of the intertemporal constriction, supraoccipital overlaps median portions of parietals and obscures them; (2) are completely absent in the skull roof; (3) visible only as triangular areas dorsolateral to supraoccipital, supraoccipital does not overlap parietals but separates and contacts them along an irregular suture.

135. *Interparietal*: (0) present; (1) absent or fused so that it is not distinguishable from parietals and frontals.

136. *Cross-section through intertemporal region, including parietals*: (0) ovoid cross-section with sagittal crest; (1) ovoid but sagittal crest absent; (2) pinched ventrally and dorsal part expanded laterally, expanded part is rounded-over in cross-section; (3) dorsal part is greatly expanded, overhangs more ventral portions, and lateral edge of dorsal surface is a sharp ridge.

137. *Length of intertemporal region, ventral view of skull roof with basicranium removed*: (0) intertemporal region absent or short, canal in frontals that contained olfactory stem and bulbs is < 10% of the length from posterior edge of dorsal nasal sinuses to posterior edge of skull; (1) long, canal is between 18% and 35% of that length; (2) very long, canal > 44% of that length.

138. *Dorsoventral thickness of intertemporal region*: (0) thin, thickness is < 25% the maximum height of skull, as measured from intercondylar notch to dorsal-most point of the supraoccipital; (1) thick, thickness between 30% and 43% of the skull height; (2) very thick, thickness > 54% of the skull height.

139. *Anterior-most point of the supraoccipital, in dorsal view*: (0) in transverse line with space between posterior edge of skull and anterior edge of the floor of squamosal fossa; (1) in line with space between anterior edge of the floor of squamosal fossa and the anterior tip of zygomatic process of squamosal; (2) in line with gap between anterior edge of zygomatic process of squamosal and the anterior-most point along posterior edge of the supraorbital process of frontal; (3) in line with supraorbital process of frontal; (4) in line with or anterior to anterior edge of supraorbital process of frontal; anterior edge of supraorbital process is taken at its medial-most point.

140. *Pronounced bulge anterior to alisphenoid exposure in temporal fossa*: (0) absent; (1) present.

141. *Alisphenoid*: (0) broadly exposed laterally in temporal fossa; (1) lateral surface is broadly overlapped by parietal so that only a narrow strip on the ventral edge of temporal fossa is visible in lateral view.

142. *Zygomatic process of squamosal*: (0) directed anteriorly; (1) directed anterolaterally.

143. *Dorsal edge of zygomatic process, skull in lateral view*: (0) gently convex dorsally; (1) near anterior end there is a distinct dorsal flange or process, flange usually articulates with frontal; (2) concave dorsally.

144. *Emargination of posterior edge of zygomatic process by sternomastoid muscle fossa, skull in lateral view*: (0) absent, posterior edge forms nearly a right angle with dorsal edge of zygomatic process of squamosal; (1) slight emargination; (2) deep emargination.

145. *Width of squamosal lateral to exoccipital, skull in posterior view*: (0) narrow, exposed portion of squamosal < 14% the distance between sagittal plane and lateral edge of exoccipital; (1) intermediate width, width between 16% and 35% of that distance; (2) wide, width between 40% and 55% of that distance; (3) very wide, width > 129% the distance between sagittal plane and lateral edge of exoccipital.

146. *Depth of squamosal fossa*: (0) absent or very shallow, depth of fossa < 52% the horizontal distance from dorsal edge of zygoma to the point above deepest part of squamosal fossa; (1) shallow, depth between 55% and 91% that distance; (2) deep, depth between 98% and 168% that distance; (3) very deep, depth greater than 180% that distance.

147. *Longitudinal profile of floor of squamosal fossa*: (0) highly sigmoidal, concave posteriorly in region of a secondary squamosal fossa (sensu Sanders and Barnes, 2002) but convex anteriorly; (1) slightly sigmoidal, posterior part concave but does not form a discrete pit; (2) flat; (3) convex.

148. *Floor of squamosal fossa*: (0) same dorsoventral thickness anteriorly and posteriorly; (1) thickens posteriorly.

149. *Squamosal prominence*: (0) absent; (1) present, forms a medial projection on crest that forms the lateral edge of squamosal fossa, is continuous with a dorsoventral ridge on lateral wall of the squamosal fossa.

150. *Ventral edge of zygomatic process of squamosal in lateral view*: (0) concave ventrally; (1) straight; (2) convex ventrally .

151. *Postglenoid process in lateral view*: (0) tapers ventrally to a point; (1) anterior and posterior sides nearly parallel with squared-off ventral end; (2) same as state 1 except anteroposterior diameter of postglenoid process is very wide.

152. *Anterior edge of supraoccipital in dorsoposterior view*: (0) triangular, pointed anteriorly; (1) semicircular; (2) rectangular.

153. *Lambdoidal crests of supraoccipital*: (0) horizontal and directed laterally, overhanging temporal fossae; (1) directed dorsolaterally, not or only slightly overhanging temporal fossae; (2) very low and not directed either way.

154. *Posteromedial wall of temporal fossa*: (0) visible in dorsal view; (1) hidden in dorsal view by lateral edges of supraoccipital.

155. *Occipital shield*: (0) smoothly convex or concave; (1) bears distinct sagittal crest.

156. *Dorsal condyloid fossa*: (0) absent; (1) present, situated anterodorsal to dorsal edge of condyle; (2) present and forms a deep pit.

157. *Anterior sinus*: (0) absent; (1) present but short; (2) elongate with corresponding trough on maxilla.

158. *Palatine*: (0) relatively thin, floors posterior part of nasal cavity; (1) thick, forms part of the anterior wall of the nasal cavities.

159. *Palatines*: (0) exposed ventrally; (1) partially covered by pterygoid dividing it into medial and lateral exposures; (2) ventral surfaces covered completely by pterygoids.

160. *Palatine*: (0) ventral surface flat or convex; (1) bears fossa for anterior end of pterygoid sinus; (2) fossa well developed, divides palatine into medial and lateral laminae .

161. *Lateral lamina of palatine*: (0) free from or sutured to maxilla; (1) fused to maxilla.

162. *Pterygoid/palatine suture in ventral view*: (0) angled anterolaterally; (1) nearly transverse, pterygoid forms a substantial part of subtemporal crest; (2) angled anteromedially.

163. *Pterygoid sinus fossa*: (0) absent or cannot be distinguished from anterior part of fossa for cavum tympani; (1) present, anterior edge approximately in line with anterior edge of foramen ovale; (2) present and extended well anterior to foramen ovale; (3) extended anterior to anterior edge of orbit.

164. *Lateral lamina (outer plate or external duplication) of pterygoid*: (0) present; (1) partial, restricted to region lateral to the hamular process; (2) absent.

165. *Subtemporal crest*: (0) present on alisphenoid and/or pterygoid, marks lateral edge of pterygoid fossa; (1) subtemporal crest absent, pterygoid fossa extended laterally into orbital region.

166. *Inferior lamina of pterygoid*: (0) absent or restricted to extreme anterior edge of pterygoid sinus cavity; (1) present and floors most of sinus cavity.

167. *Superior lamina of pterygoid*: (0) present and covers most of ventral exposure of alisphenoid; (1) absent from the sphenoidal region but present in orbital region; (2) partially absent from orbital region; (3) completely absent from orbital region.

168. *Posterior part of pterygoid sinus fossa, region immediately anterior to exit for mandibular branch of trigeminal nerve*: (0) one single fossa; (1) split into a smaller and shallower posterior fossa and a much larger anteriorly extended deeper fossa by a low ridge; (2) same as state 1 except divided by a high ridge .

169. *Preorbital lobe of pterygoid sinus*: (0) present; (1) present but small; (2) present and enlarged; (3) enlarged and forms distinct excavation anterior to optic foramen; (4) enlarged and extended posterodorsally over frontals to be roofed by maxilla.

170. *Postorbital lobe of pterygoid sinus*: (0) absent; (1) present but small; (2) present and enlarged; (3) enlarged and forms prominent fossa on ventral surface of supraorbital process of frontal posterior to optic foramen.

171. *Coalescence of pre-orbital and post-orbital lobes of pterygoid sinus dorsal to optic nerve*: (0) absent; (1) present.

172. *Hamular process of the pterygoid*: (0) splint like; (1) solid, long and subconical; (2) hollow and excavated by pterygoid sinus, lateral side highly concave, may or may not have lateral and inferior laminae; (3) form thin horizontal plates; (4) absent.

173. *Hamular processes of pterygoids*: (0) rounded over in ventral view; (1) bear anteroposterior keels.

174. *Posterior-most point of hamular process of pterygoid, or medial part of pterygoid if the hamular process is absent*: (0) in transverse line with the middle of orbit; (1) in line with postorbital process; (2) in line with anterior edge of zygomatic process of squamosal; (3) in line with middle of zygomatic process; (4) in line with the postglenoid process.

175. *Fossa for pterygoid sinus on alisphenoid posterior to groove for mandibular branch of trigeminal nerve*: (0) absent, bone is flat or not ossified because of enlarged internal foramen ovale; (1) shallow fossa; (2) deep subcircular fossa.

176. *Falciform process of squamosal*: (0) plate-like with a wide, anteroposteriorly, base; (1) rod-like with narrow base; (2) poorly-developed or absent.

177. *Falciform process of the squamosal*: (0) medial surface sutured to lateral lamina of pterygoid; (1) not sutured.

178. *Tympanosquamosal recess*: (0) absent; (1) absent but a small rectangular fossa for sigmoid process of the tympanic present, its long axis is transverse, and it is located medial to postglenoid process of squamosal; (2) present and enlarged, forms a triangular fossa medial and anteromedial to postglenoid process; (3) very large, forms large fossa that borders entire medial edge of glenoid fossa.

179. *Lateral edge of middle sinus*: (0) smooth; (1) deckle-edged.

180. *Position of alisphenoid/squamosal suture, skull in ventral view*: (0) anterior to external foramen of foramen ovale or homologous groove; (1) courses along groove for mandibular branch of trigeminal nerve, or just posterior to it; (2) just medial to anterior edge of floor of squamosal fossa, foramen ovale, and/or groove situated entirely on alisphenoid.

181. *Groove for mandibular branch of trigeminal nerve*: (0) directed laterally and is entirely posterior to pterygoid sinus fossa; (1) lateral end of groove wraps laterally around the posterior end of the pterygoid sinus fossa and opens primarily anteriorly.

182. *Ventral part of squamosal posterior to postmeatic process*: (0) large area of laminated bone, appears externally as multiple sutures; (1) small area of laminated bone restricted to ventrolateral edge of squamosal; (2) without laminated bone.

183. *Cranial hiatus*: (0) absent, petrosal contacts basioccipital medially or partially separated by narrow fissure; (1) present but constricted, a medial projection of parietal partially divides fenestra; (2) absent, parietal contacts basisphenoid and/or basioccipital dividing fenestra in two; (3) present, wide space between basioccipital and both petrosal and squamosal.

184. *Periotic fossa*: (0) bowl-shaped; has transverse ridge that divides it into anterior and posterior portions.

185. *Suprameatal pit of squamosal*: (0) absent; (1) present but shallow, situated dorsolateral to spiny process of squamosal; (2) forms a deep dorsolateral excavation into squamosal.

186. *Foramen spinosum*: (0) absent; (1) present, located in anteromedial corner of anterior part of periotic fossa near or on squamosal/parietal suture.

187. *Posterior portion of periotic fossa*: (0) posteromedial part contains a deep, large fossa; (1) fossa present but shallow; (2) fossa is highly compressed and forms a narrow slit or a small blind foramen; (3) fossa absent, posterior portion of the periotic fossa is of uniform depth.

188. *Zygomatic process of squamosal*: (0) very short, length of process < 92% of the maximum width of glenoid fossa; (1) short, length between 103% and 162% of glenoid width; (2) intermediate length, length between 171% and 189% of the glenoid width; (3) long, length between 198% and 271% of glenoid width; (4) very long, length > 300% of the width of glenoid fossa.

189. *External auditory meatus*: (0) wide; (1) narrow.

190. *Vomer*: (0) posterior edge terminates on or at anterior edge of basisphenoid; (1) terminates on basioccipital covering basioccipital/basisphenoid suture ventrally.

191. *Basioccipital crest*: (0) narrow transversely; (1) wide and bulbous.

192. *Rectus capitus anticus muscle fossa*: (0) absent or poorly developed; (1) present with a well-defined anterior edge.

193. *Posteroventralmost point of basioccipital crest*: (0) rounded over; (1) forms a closely appressed separate flange, a narrow crease separates it dorsally from rest of basioccipital crest; (1) distinct flange that projects posteriorly; (2) distinct but separated by a pronounced notch that interrupts basioccipital crest .

194. *Angle formed by the basioccipital crests in ventral view*: (0) parallel with no angle formed; (1) 15° to 40°; (2) 45° to 68°; (3) 74° to 90°; (4) > 100°.

195. *Hypoglossal foramen*: (0) thick bone separating it from jugular foramen, or jugular notch; (1) separating bone very thin or absent, in the latter case hypoglossal foramen becomes confluent with jugular foramen.

196. *Jugular notch, gap between paroccipital process and basioccipital crest*: (0) open notch, opening and depth of the notch are roughly equal; (1) narrow and almost slit-like, depth is much greater than width of opening.

197. *Paroccipital process, skull in ventral view*: (0) angled posterolaterally, extends posterior to posterior-most edge of condyle; (1) posterior edge in transverse line with posterior edge of condyle; (2) posterior edge is well anterior to posterior edge of condyle .

198. *Tuberculum of the malleus*: (0) unreduced; (1) highly reduced, almost indistinguishable from articular head .

199. *Processus muscularis of malleus*: (0) processus muscularis shorter than manubrium of malleus; (1) subequal; (2) processes muscularis longer than manubrium.

200. *Apex of anterior process of the petrosal*: (0) in ventral or dorsal view blunt or pointed; (1) bears tubercle.

201. *Anterior process in lateral view*: (0) anterior edge of anterior process squared-off; (1) Comes to a blunt apex; (2) comes to a slender point.

202. *Apex of anterior process of the petrosal*: (0) at same level or dorsal to ventral edge of pars cochlearis; (1) well ventral to ventral edge of the pars cochlearis, process appears to be ventrally deflected.

203. *Length of anterior process of the petrosal*: (0) absent; (1) present but very short, length < 36% of the length of the pars cochlearis; (2) short, length between 59% and 94% of the promontorial length; (3) nearly the same as length of pars cochlearis, length between 100% and 134% of the promontorial length; (4) long, length between 141% and 174% of the promontorial length; (5) very long, length > 212% of the length of the pars cochlearis.

204. *Anterior process in lateral view*: (0) ventral edge convex ventrally or nearly flat; (1) ventral edge clearly concave.

205. *Anteroexternal sulcus*: (0) absent; (1) present on lateral surface of anterior process of petrosal, oriented primarily anteroposteriorly but bowed ventrally.

206. *Sulcus for capsuloparietal emissary vein*: (0) present, forms a dorsoventral groove on lateral side of anterior process immediately anterior to lateral tuberosity; (1) Absent.

207. *Articulation of anterior process with squamosal*: (0) extensive, most of lateral side contacts squamosal; (1) large centrally-oriented ovoid region contacts squamosal, free around the edges; (2) contact is very small; (3) contact is absent, articulates via ligaments.

208. *Shape of cross section through anterior process at midlength*: (0) highly elliptical, transverse diameter is < 36% the dorsoventral diameter; (1) ovoid, transverse diameter is between 51% and 78% of the dorsoventral diameter; (2) approximately circular, transverse diameter between 85% and 134% of the dorsoventral diameter.

209. *Contact of anterior process of petrosal with portion of ectotympanic bulla anterior to accessory ossicle*: (0) absent; (1) present but no clear fossa for articulation on petrosal; (2) anterior bullar facet present but shallow with poorly defined medial edge; (3) present with well defined medial and lateral edges.

210. *Flange of anterior process of petrosal*: (0) absent; (1) present.

211. *Lateral tuberosity*: (0) absent; (1) present, forms a bulbous prominence lateral to fossa for malleus; (2) present and elongate, forms a lateral process that articulates dorsally with squamosal.

212. *Emargination of lateral edge of petrosal by hiatus epitympanicus*: (0) with petrosal in ventral view, emargination is narrow and is situated slightly posterior to base of posterior process; (1) emargination is wide and is approximately in line with gap between fenestrae ovalis and rotunda .

213. *Fossa incudes*: (0) poorly defined or cannot be differentiated from rest of epitympanic recess; (1) forms a clear circular fossa; (2) circular fossa present on a short pedestal, the incudal process.

214. *Fossa for malleus*: (0) present; (1) absent or poorly developed.

215. *Ventrolateral ridge of petrosal*: (0) absent; (1) present; (2) present and expanded.

216. *Lateral side of petrosal*: (0) entire side of petrosal contains pitted and rugose bone; (1) all but anterior process is rugose; (2) lateral side of posterior process of petrosal is pitted and rugose, remaining portions are smooth; (3) entire side of petrosal is smooth.

217. *Origin of tensor tympani muscle*: (0) deep, pocket-like fossa with anterior groove; (1) anterior groove only; (2) broad, poorly defined origin without a clear groove.

218. *Angle between anterior process of petrosal and anterior edge of pars cochlearis*: (0) obtuse, pars cochlearis appears transversely compressed; (1) nearly 90 degrees, pars cochlearis looks rectangular or semicircular in ventral view; (2) acute, pars cochlearis looks globular.

219. *Anteromedial corner of pars cochlearis*: (0) Rounded; (1) angular.

220. *Pars cochlearis*: (0) most convex part is on ventrolateral surface most convex part is on medial surface; (1) area of greatest convexity begins anteromedial to fenestra rotunda and extends anterodorsally on the medial face, with petrosal in dorsal view, there is a wide expanse of bone medial to internal acoustic meatus .

221. *Ridge on anterolateral side of pars cochlearis, petrosal in ventral view*: (0) present and high, forms an anteroposterior ridge that also forms the medial edge of a trough for tensor tympani muscle; (1) present and low; (2) absent.

222. *Fenestra rotunda*: (0) oval; (1) shaped like a teardrop with a fissure directed towards the perilymphatic foramen.

223. *Posterodorsal edge of stapedial muscle fossa*: (0) ventral to or in line with dorsal edge of fenestra rotunda; (1) well dorsal to fenestra rotunda.

224. *Stylomastoid fossa*: (0) absent; (1) present, situated on posterior face of pars cochlearis posterodorsal to stapedial muscle fossa; (2) enlarged dorsally and medially, covers much of posterior face of pars cochlearis; (3) enlarged posterolaterally onto posterior process of the petrosal.

225. *Caudal tympanic process of the petrosal*: (0) prominent, its ventral and posterior edges form a right angle in medial view; (1) low, its ventral and posterior edges are joined by a smooth curve .

226. *Caudal tympanic process of petrosal in posteromedial view*: (0) well separated from crista parotica, no division between stapedial muscle fossa and stylomastoid foramen; (1) narrow separation or contact, clear separation of stapedial muscle fossa and stylomastoid foramen.

227. *Perilymphatic foramen*: (0) smaller than endolymphatic foramen; (1) approximately the same size much larger with narrow posterior edge.

228. *Distance between perilymphatic foramen and fenestra rotunda*: (0) no distance, both apertures are confluent; (1) narrow, distance < 89% of the distance between fenestra ovalis and fenestra rotunda; (2) narrow, distance > 89% of the distance between fenestra ovalis and fenestra rotunda; (3) very wide, distance > 146%.

229. *Distance between endolymphatic foramen and fenestra rotunda*: (0) very narrow, distance < 112% of the distance between fenestra ovalis and fenestra rotunda; (1) narrow, distance between 121% and 185%; (2) wide, distance between 192% and 211%; (3) very wide, distance > 222% the distance between fenestra ovalis and fenestra rotunda.

230. *Elongation of pars cochlearis towards cranial cavity, dorsally and medially*: (0) absent; (1) present, inner porous bone expanded towards cranial cavity; (2) present, outer periosteal bone of pars cochlearis expanded towards cranial cavity.

231. *Excavation of tegmen tympani at base of anterior process*: (0) absent; (1) present, fossa on dorsolateral side of tegmen tympani.

232. *Dorsal edge of tegmen tympani dorsolateral to internal acoustic meatus and anterior process*: (0) present and high, dorsoventral height > 114% the width of pars cochlearis; (1) present, height between 58% and 34% of promontorial width; (2) low, height between 23% and 11%; (3) forms a low ridge or is absent, height < 4% of the width of pars cochlearis.

233. *Dorsal edge of tegmen tympani lateral to endolymphatic foramen*: (0) present and very high, dorsoventral height > 112% of the width of pars cochlearis; (1) high, height between 95% and 50% the promontorial width; (2) low, height between 12% and 4%; (3) faint ridge; (4) absent.

234. *Fundus of internal acoustic meatus*: (0) funnel-like, smaller at the blind end and wider near the rim; (1) tubular.

235. *Lateral wall of internal acoustic meatus*: (0) low, does not protrude noticeably from suprameatal fossa and surrounding bone; (1) high, a wedge-shaped area of elevated bone occurs between dorsal edge of tegmen tympani and internal acoustic meatus, extending the latter ventrally and increasing its depth.

236. *Foramen singulare*: (0) in common recess with the tractus spiralis foraminosus, transverse septum separating the foramen singulare from endocranial aperture of facial nerve is well developed; (1) in common recess with tractus spiralis foraminosus, transverse septum separating it from facial nerve foramen is low, and endocranial aperture of facial nerve canal within internal acoustic meatus; (2) separated by partitions of equal height from tractus spiralis foraminosus and endocranial aperture of facial nerve canal; (3) in common recess with endocranial aperture of facial nerve canal.

237. *Endocranial aperture of facial nerve canal*: (0) anterior to tractus spiralis foraminosus; (1) slightly anterior, posterior edge of aperture of facial nerve canal is lateral to center of tractus spiralis foraminosus; (2) lateral to tractus spiralis foraminosus.

238. *Morphology of endocranial opening of facial nerve canal*: (0) continuous with an anterior fissure; (1) oval-shaped; (2) circular.

239. *Articular rim*: (0) absent; (1) present but small, forms a ridge anterolateral to articulation surface of the posterior process of the petrosal and separated from it by a sulcus; the ridge fits into a corresponding cavity posterolateral and slightly dorsal to spiny process of squamosal; (2) present, long, oriented posterodorsally, and posterior end intersects dorsal margin of petrosal; in lateral view has sigmoidal shape; (3) present with sigmoidal shape and laterally elongate with hook-like process.

240. *Contact of petrosal, not including anterior process, with skull*: (0) distal end of posterior process of petrosal, lateral surface of posterior process of the petrosal, and entire dorsal edge of tegmen tympani (or homologous bone) contact squamosal and possibly parietal; (1) same as state “0” except dorsal edge contacts from posterior end of posterior process of petrosal to region just lateral to endolymphatic foramen; (2) only dorsal and lateral sides of posterior process articulate with squamosal; (3) petrosal articulates with squamosal along hiatus epitympanicus and adjacent regions on the posterior process; (4) petrosal only articulates with skull via ligaments.

241. *Articulation surfaces on posterior processes of ectotympanic and petrosal*: (0) surfaces smooth; (1) bear complimentary longitudinal grooves and ridges; (2) fused in adults.

242. *Ventral surface of posterior process of petrosal, along a straight path perpendicular to its long axis*: (0) concave; (1) flat; (2) convex.

243. *Bullar facet on posterior process of petrosal*: (0) restricted to ventral surface; (1) extends dorsally onto posteromedial face of posterior process.

244. *Facial nerve sulcus*: (0) long sulcus on posterior process of petrosal or the compound petrosal/tympanic posterior process of most mysticetes; (1) short, no sulcus posterior to stylomastoid notch.

245. *Length of posterior process of petrosal*: (0) absent or very short, length < 47% the length of pars cochlearis; (1) short, length between 88% and 119% of the promontorial length; (2) slightly longer than pars cochlearis, length between 131% and 153%; (3) long, length between 191% and 404%; (4) very long, length > 613% of the length of pars cochlearis.

246. *Orientation of posterior process of petrosal*: (0) forms an angle < 130° with the long axis of tegmen tympani; (1) forms an angle between 135° and 165°; (2) directed nearly posteriorly, forms an 180° with tegmen tympani.

247. *Dorsal edge of posterior process, petrosal in lateral or medial view*: (0) straight or convex ventrally; (1) concave ventrally, helps to form the neck of posterior process of petrosal .

248. *Posterior process of petrosal*: (0) robust; (1) horizontal plate and very thin for most of its length.

249. *Mastoid exposure of posterior process of petrosal on the outside of skull*: (0) exposed externally; (1) not exposed, enclosed by the exoccipital and squamosal.

250. *Anterior spine, or conical anterior tip, of ectotympanic bulla*: (0) absent; (1) present but small; (2) present and long.

251. *Shape of ectotympanic bulla*: (0) narrow and long, width of bulla at sigmoid process is < 64% the length of bulla along its long axis; (1) wide, width of bulla > 65% of its long axis.

252. *Posterior end of ventromedial keel*: (0) forms a smooth curve around posterior part of involucrum; (1) protrudes and points medially.

253. *Accessory ossicle*: (0) absent; (1) present.

254. *Accessory ossicle*: (0) small and oblong; (1) large and subspherical.

255. *accessory ossicle or homologous region on lip of bulla*: (0) fused to anterior process of petrosal; (1) not fused.

256. *Lateral furrow of ectotympanic bulla*: (0) present; (1) absent.

257. *Lateral furrow*: (0) broad sulcus; (1) narrow crease.

258. *Sigmoid process*: (0) forms a straight transverse plate that is directed perpendicular to long axis of bulla; (1) forms a curved plate; proximal part is directed posterolaterally while the distal end curves to point laterally.

259. *Ventral margin of sigmoid process*: (0) present; (1) absent; in its place the lateral margin of sigmoid process smoothly turns into a sulcus on lateral side of bulla.

260. *Dorsal edge of sigmoid process*: (0) contacts sigmoid fossa of squamosal; (1) distal end expanded anteriorly to articulate with lateral tuberosity of petrosal only; (2) does not articulate with squamosal or petrosal.

261. *Elliptical foramen of ectotympanic bulla*: (0) present, connection between ectotympanic bulla and its posterior process is split into two pedicles; (1) absent.

262. *Anterior edge of posterior process of ectotympanic bulla*: (0) contacts postmeatic process of squamosal; (1) contact absence.

263. *Posterior process of tympanic*: (0) contains sporadic areas of laminated bone at posterodorsal end; (1) almost entire process is laminated bone.

264. *Thickness of posterior process of ectotympanic*: (0) thick in region ventral to articulating surface with petrosal; (1) forms a thin lamina.

265. *Distal end of posterior process of ectotympanic*: (0) thinner or approximately the same thickness as more proximal portions; (1) distal end thicker but not hypertrophied; (2) hypertrophied in size, forms large nodular mass.

266. *Median furrow of ectotympanic bulla*: (0) absent; (1) forms notch on posterior edge of bulla between medial and lateral prominences; (2) forms continuous anteroposterior groove on ventral surface of bulla.

267. *Median furrow*: (0) on posterior side of bulla, it is divided by a transverse ridge originating from the involucrum; (1) transverse ridge is absent.

268. *Profile of ectotympanic bulla in lateral view*: (0) ventral edge convex or flat; (1) concave because of posteroventral expansion of lateral prominence.

269. *Medial prominence of involucrum*: (0) posterior edge approximately in line with posterior edge of lateral prominence; (1) posterior edge distinctly anterior to posterior edge of lateral prominence.

270. *Involucrum*: (0) in medial view, dorsal and ventral borders converge anteriorly; (1) excavated anterior to base of posterior process so that dorsal and ventral sides are parallel.

271. *Involucrum*: (0) bears prominent transverse groove on dorsal surface that divides involucrum into a thicker posterior part and thinner anterior part; (1) groove absent.

272. *Ridge on inside of bulla*: (0) present, transverse ridge extends laterally from involucrum and partially divides cavum tympani into anterior and posterior portions; (1) absent.

273. *Ventromedial keel of the ectotympanic bulla*: (0) present along entire length; (1) terminates approximately at level of lateral furrow; (2) poorly defined along entire length.

274. *Shape of ventromedial keel, bulla in dorsomedial view*: (0) nearly straight; (1) bowed medially.

275. *Region on dorsomedial side of ventromedial keel*: (0) flat or convex; (1) gently concave.

276. *Length of prelumbar vertebral column*: (0) very short, length of cervical plus thoracic portions of vertebral column from 77% to 55% condylobasal length of skull; (1) short, length of cervical plus thoracic portions of vertebral column from 100 to 83% skull length; (2) long, from 123% to 114% skull length; (3) very long, > 135% skull length.

277. *Atlas*: (0) ventral process larger than dorsal process; (1) both processes are subequal; (2) dorsal process larger.

278. Atlas and axis vertebrae: (0) unfused; (1) fused together.

279. *Cervical vertebrae posterior to atlas*: (0) all are separate; (1) only 2nd and 3rd are fused together; (2) 2nd through 4th are fused together; (3) 2nd through 5th are fused; (4) 2nd through 6th are fused; (5) 2nd through 7th are fused.

280. *Number of thoracic vertebrae*: (0) 18 to 17; (1) 16 to 15; (2) 14; (3) 13; (4) 12; (5) 11; (6) 10 or less.

281. *Number of thoracic vertebrae with capitular articulations*: (0) 11; (1) 10; (2) 9; (3) 8; (4) 7; (5) 6; (6) 4 to 5; (7) 3 or less.

282. *Capitular articulation facets of the posterior vertebrae*: (0) facets gradually shift downward on sequential vertebrae to fuse with the tubercular facets; (1) facets abruptly shift from a position on the neural arch to a pedestal that originates from the centrum on the subsequent vertebra.

283. *Lateral edge of transverse processes of lumbar vertebrae*: (0) oriented anteroposteriorly; (1) angled anteromedially 45° or more, relative to a parasagittal plane.

284. *Transverse processes of lumbar vertebrae*: (0) oriented ventrolaterally; (1) oriented laterally and horizontally.

285. *Lumbar vertebrae*: (0) transverse processes narrow distally or are approximately the same anteroposterior width as their bases; (1) transverse processes bear greatly expanded distal ends.

286. *Centrum of anterior lumbar vertebrae*: (0) short, length < 63% the width; (1) long, length between 79% and 136% the width; (2) very long, length > 147% the width.

287. *Number of lumbar vertebrae*: (0) 3 or less; (1) 4; (2) 6; (3) 7 to 8; (4) 9 to 10; (5) 11 to 12; (6) 13 to 16; (7) 16 to 19.

288. *Number of caudal vertebrae*: (0) 13 to 15; (1) 16 to 19; (2) 20 to 23; (3) 24 to 27; (4) 27 to 30; (5) 33 to 33; (6) 34 to 60.

289*. Ventrolateral processes on manubrium of sternum*: (0) absent; (1) present but small, occurs ventral to articulation surface for first costal cartilage or rib.

290. *Sternum*: (0) comprised of several bones: (1) comprised of one bone.

291. *Sternum*: (0) several ribs attach to sternum; (1) one rib attaches to sternum.

292. *Coracoid process of scapula*: (0) present; (1) absent or barely indistinguishable from edge of glenoid fossa.

293. *Supraspinous fossa of scapula*: (0) present; (1) absent or nearly absent, acromion process on anterior edge of scapula.

294. *Prominent deltoid crest on anterior edge of humerus*: (0) present, forms greatest anteroposterior diameter along shaft; (1) forms a knob-like tuberosity; (2) tuberosity and crest absent.

295. *Delto-pectoral tuberosity or farthest anterior point of crest*: (0) closer to proximal head of humerus; (1) approximately centered, proximodistally, on shaft; (2) closer to distal end of humerus.

296. *Radial and ulnar facets of humerus*: (0) forms one articulation surface that is semicircular in lateral view; (1) two distinct facets that in lateral view form an obtuse angle.

297. *Humerus*: (0) longer than radius and ulna; (0) approximately the same length as radius and ulna; (1) shorter than radius and ulna.

298. *Olecranon process*: (0) present as a distinct process; (1) present as slightly raised proximal posterior edge; (2) absent.

299. *Manus*: (0) pentadactyl; (1) tetradactyl.

300. *Esophageal forestomach*: (0) present, epithelium is either completely devoid of glands or may contain a small patch; (1) absent, first chamber of stomach has glandular epithelium.

301. *External throat grooves*: (0) absent; (1) one pair, one on each side of midline; (2) one to five pairs of grooves; (3) more than five pairs.

302. *Throat grooves*: (0) convergent anteriorly; (1) parallel.

303. *Sexual dimorphism*: (0) males 45% larger than females; (1) males 14% to 30% larger than females; (2) females and males approximately the same size; (3) females 5% to 10% larger than males; (4) females 20% to 30% larger than males.

304. *Dorsal fin*: (0) absent; (1) dorsal hump; (2) present.

305. *Dorsal margin of mesethmoid*: (0) below level of adjacent premaxilla; (1) flush with or nearly flush with premaxilla; (2) distinctly above level of adjacent premaxilla.

306. *Straight line distance between posterior-most point of right premaxilla along opening of bony external nares and the right nasal*: (0) right premaxilla and nasal contact each other or are separated by a narrow gap that is < 15% of the maximum width of the external bony nares; (1) intermediate separation, distance is between 30 and 50% of maximum nares width; (2) wide, distance is > 60% of maximum nares width.

307. *Orientation of the medial portion maxilla that is situated on either side of the vertex*: (0) faces mainly laterally faces mainly dorsolaterally; (1) faces mainly dorsally; (2) faces mainly anterodorsally.

308. *Deep wedge of supraoccipital between frontals on vertex*: (0) absent; (1) present.

309. *Lateral margin of posterolateral sulcus*: (0) low; (1) high and as a result posterolateral sulcus is deeply entrenched; (2) high, but posterolatral sulcus is smooth, not entrenched.

310. *Deep sagittal sulcus on middle of occiput*: (0) absent; (1) present.

311. *Longest side of nasal faces*: (0) dorsally; (1) mainly anterodorsal; (2) mainly anteriorly.

**2. Character-Taxon Matrix**

The matrix used here is based on that published by Aguirre and Fordyce (2014) with the addition of ***Isthminia panamensis*** and updated scoring for *Ischyrorhynchus vanbenedeni* (updated characters marked in bold). The matrix includes 2 outgoup and 50 ingroup taxa; and 311 multistate characters, all characters were left unordered and of equal weight.

*Georgiacetus vogtlensis*

? 0 0 0 0 0 1 0 0/1 2 0 0 0 ? 0 0 0 0 0 0 ? 0 5 1 0 1 ? ? 0 1 1 0 1 ? ? ? 3/4 0 1 1 0 0/1 1 1 0 0 2 ? 5 ? 0 0 0 0 ? 0 0 ? 2 2 1 0 0 0 0 ? 2 1 0 ? ? ? ? 0 ? 0 1 ? ? 1 0 0 0 0 1 ? ? 0 ? 0 0 0 ? ? ? ? ? ? ? ? ? ? ? ? ? ? 0 0 0 0 ? 0 0 1 0 0 0 0 2 2 0 0 0 2 ? 0 ? 2 0 ? 1 ? 0 0 ? 0 1/2 2 0 0 0 1 0 ? 0 2/3 2 ? 1 0 0 1 1 ? ? 0 0/1 0 0 0 ? 2 1 ? 0 0 1 ? 0/1/2 0/1/2 ? 4 ? 2 1 0 1 0 ? 0 1 0 3 ? ? ? 3 1 0 ? 0 0 0 0 0 0 2 ? ? 0 ? ? 1/2 0 ? 0 0 1/2 ? 0 0 ? ? 0 0 ? 0 0 1 0 2 0 ? ? ? ? ? ? ? 0 ? ? ? ? 1 ? ? ? ? ? ? ? ? ? 3 0 ? 0 0 0 1 0 ? ? 1 0 ? ? ? ? 0 0 1 0 1 2 ? 0 0 0 ? ? ? 0 0 ? ? ? 0 2/3/4 0/1 ? 0 0 0 0 3/4 ? ? ? ? ? ? ? ? ? ? ? ? ? ? ? ? ? ? ? ? ? ? ? ?

*Delphinus*

0 0 1 0 0 0 2 0 1 0 1 0 0 ? 1 0 0 1 1 1 0 0 0 6 0 0 0 0 0 0 0 1 1 ? 1 0 9 0 0 1 0 1 1 0 1 0 1 0 2 ? 1 1 1 ? 1/2 1 0 0 0 ? 1 0 0 2 0 0 2 0 1 0 1 0 0 4 1 2 5 0 0 5 1 1/2 0 1 2 ? 0 0 0 0 0 1 1 0 2 1 0 2 1 0 1 ? 2 1 ? 1 ? ? 0 0 0 1 0 2 ? 0 0 0 1 1 0/1 4 4 1 2 0 0 1 0 0 0 0 0 3 0 3 0 0 1 0 0 1 1 2 1 1 2 0 0 0 1 2 1 ? 0/1 1 2 1 0 2 0 2 3 1 1 0 2 0 4 3 1 2 1 0 1 0 ? 3 1 2 ? 1 2 0 0 ? 3 1 1 1 0 0 0 2 0 1 2 0 1 0 0 1 2 1 0 1 3 2 ? 0 1 1 0 0 1 3 1 1 0 1 2 0 1 0 1 0 1 2 1 0 0 3 4 0 0 2 0 2 0 3 1 0 0 0 1 1 0 0 1 0 0 0 1 0 1 1 ? 0 0 1 0 0 0 1 0 2 1 1 1 1 1 1 1 0 0 1 0 1 0 2/3 1 0 0 1 0 1 7 4 0 ? 0 0 0 1 2 1 2 0 1 1 0 ? 2 2 ? ? ? ? ? ? ?

*Tursiops truncatus*

0 0 1 0 0 0 2 0 2 2 1 0 0 ? 1 0 0 1 0 1 0 0 0 5 0 0 0 0 0 0 0 1 1 ? 0 0 6/7 0 0 1 0 1 1 0 0 0 1 0 2 ? ? 1 1 ? 1 1 1 0 0 ? 0 1 0 0/1 0 1 2 0 1 0 1 1 0 2 1 2 4 1 0 5 1 1 0 1 2 ? 0 0 1 1 0 1 1 0 2 1 0 2 1 0 1 1 2 1 1 1 ? ? 0 0 0 1 0 2 ? 0 0 0 1 3 1 3 3 1 ? 0 0 1 0 0 0 0 0 3 0 3 0 0 0 0 ? 1 1 2 1 1 2 0 0 0 1 2 1 ? 0/1 1 2 1 0 2 0 2 ? 1 1 0 2/3 0 3 3 1 2 1 1 1 0 ? 3 1 2 0 1 2/3 0 0 0/1 0/1 1 1 1 0 0 0 3 0 1 2 0 2 0 0 1 2 ? 0 1 3 1 ? 0 1 1 1 0 1 3 1 2 0 1 2 0 1 0 1 1 0 3 1 0 0 3 2 0 0 2 1 ? 0 2 1 0 0 0 1 1 0 0 1 0 0 1 1 0 1 1 ? 0 0 1 0 0 1 0/1 1 2 1 1 0 1 1 1 0/2 0 0 1 0 1 0 2/3 1/2 0 0 1 0 1 7 3/4 0 1 0 0 0 1 2 1 2 0 1 1 0 ? 2 2 ? ? ? ? ? ? ?

*Leucopleurus acutus*

0 0 1 0 0 0 2 0 3 2 1 0 0 ? 1 0 0 1/2 0 1 ? 1 0 7 0 0 0 0 1 0 0 1 1 ? ? 0 8/9 0 0 1 0 1 1 0 ? 1 1 0 0/1 1 0 1 1 ? 0 1 0 1 0 ? 1 1 0 2 0 1 2 0 1 0 1 1 0 4 1 2 5 1 0 5 1 2 0 1 2 ? 0 0 0/1 0 0 1 1 ? 2 1 0 2 1 ? ? ? 2 ? 1 1 ? ? 0 0 0 1 0 2 ? 0 0 0 2 2/3 1 5 4 1 0 0 0 0/1 0 0 0 0 0 3 ? 3 0 0 0 0 0 ? 1 1 0 2 2 1 0 0 0/1 2 0 ? 0 1 1 1 0 2 0 2 3 1 0 0 2 0 3 3 0 3 1 0 0 0 ? 3 ? 2 0 1 2 0/1 1/2 0 0/3 1 1 1 0 0 0 2 0 1 2 ? ? 0 0 1 2 1 0 0 3 ? ? 0 1 1 1 0 0 3 1 1/2 0 1 2 0/1 1 0 1 0 0 2 1 0 0 2/3 2/3/4 0 0 2 0 1 0 3/4 1 0 0 0 1 ? 0 0 1 0 0 0 1 0 1 1 ? 1 0 1 0 0 0 1 0 2 1 1 1 1 1 1 2 0 0 ? 0 1 2 1/2 6 0 0 1 0 0 7 6 1 0 0 0 0 1 2 1 2 0 1 ? ? ? ? ? ? ? ? ? ? ? ?

*Globicephala*

0 0 1 0 0 0 2 1 3 1/2 1 0 0 ? 0/1 0 0/2 1/2 0 1 0 0 0 1 0 0 0 0 1 0/1 0 1 1 ? 0 1 3 0 0 0 0 1 1 ? 0 1 1 0 1 1 1 1 1 0 1 1 0 2 0 ? 0/1 1 0 2 0 1 2 0 1 0 1 0 0 5 1/2 2 5 0/1 0 7 1 1 0 1 2 ? 0 0 0 0 0 1 1 ? 2 1 0 2 1 0 1 1 2 1 1 1 ? ? 0 1 ? 0 0 2 ? 0 0 0 2 1 1 5 4 0 ? 0 ? 0 0 0 0 0 0 3 ? 3 0 ? 0 0 0 1 0 2 0 0 2 1 0 1 0 1 1 ? 1 0 1 1 0 2 0 2 2 1 0 0 2 0 3 2 0 3 1 1 0/1 0 ? 3 0 2 0 1 2 0 0 ? 3 3 1 1 0 1 0 2 0 0/1 2 ? ? 0 0 0/1 2 0/1 0 1 2/3 1 ? 0 1 1 1 0 0 3 1 2 0 1 2 0 1 0 1 0 0/1 3 3 0 0 2 2 0 0 2 0 1/2 0 3/4 1 1 0 0 1 1 0 0 1 1 0 1 1 0 1 1 ? 1 0 1 1 0 1 1 0 2 1 1 1 1 1 1 1 0 0 ? 0 1 4/5 6 4/5/6 0 0 1 0 1 5/6 2/3/4 0 0 0 0 0 1 2 1 1 0 0 ? 0 ? 1 2 ? ? ? ? ? ? ?

*Grampus griseus*

0 0 1 1 0 0 2 0 3 2 2 0 0 ? 0 0 0 1 0 1 0 ? ? 0 ? 0 0 1 1 ? ? ? ? ? 1 1 2/3 0 0 0/1 0 1 1 0 0 1 1 0 0/1 1 0 1 1 ? 0 1 0 1 0 ? 0 1 0 2 0 1 2 0 1 0 1 1 0 5 1/2 2 5 1 1 7 1 1 0 1 2 ? 0 0 0/1 0 0 1 1 ? 2 1 0 2 1 0 ? 1 2 ? 1 1 ? ? 0 1 0 0 0 2 ? 0 0 0 2 1 1 5 4 0 0 1 ? 0 0 0 0 0 0 3 0 3 0 ? 0 0 ? 1 0 2 ? 1 2 1 0 0 1 2 1 ? 1 0 2 1 0 2 0 2 3 1 0 0 2 0 3 3 0 2 1 1 0 0 ? 3 1 2 0 1 2 0/1 0 0 0/1/3 3 1 1 0 0 0 2 0 1 2 ? ? 0 0/2 0 2 1 0 1 3 1 ? 0 1 1 1 0 0 0/1/2 1 2 0 1 2 0 1 0/1 0 1 1 3 2/3 0 0 3 4 0 0 1/2 1 1/2 0 3/4 1 0 0 0 1 2 0 0 1 1/2 0 1 1 0 1 1 ? 1 0 1 1 ? 1 1 0 2 1 1 1 1 1 1 0 0 0 ? 0 1 5 4 4/5 0 0 1 0 0/1 7 5 0 0 0 0 0 1 2 1 1 0 0 ? 0 ? ? 2 ? ? ? ? ? ? ?

*Pseudorca crassidens*

0 0 1 0 0 0 2 0/1 3 2 1 0 0 ? 0 0 0 1 0 1 0 0 0 1/2 0 0 0 0 1 0 0 1 1 ? 1 0 3 0 0 1 0 1 1 1 0 0 2 0 1 1 0/1 1 1 ? 0 1 0 2 0 ? 0 0 0 2 0 1 2 0 1 0 1 1 0 4 1/2 2 5 0/1 0 7 1 1/2 0 1 2 ? 0 0 0 1 0 1 1 ? 2 1 0 2 1 0 1 1 2 ? 1 1 ? ? 0 0/1 0 1 0 2 ? 0 0 0 2 2 1 4 4 1 0 0 0 0/1 0 0 0 0 0 2/3 ? 3 0 ? 0 0 ? 0 0 1 0/1 1 2 0 0 0 0 2 1 ? 1 0 1 1 0 2 0 2 2 1 0 0 3 0 3 1 0 3 0 2 0 0 ? 3 0 1 0 1 2 0 0 0 3 3 1 1 0 0 0 2 0 1 2 ? ? 0 0 0 2 0 0 1 3 1 ? 0 1 1 1 0 0 3 1 2 0 1 2 0 0/1 1 1 0 0 2/3 2 0 0 3 2 0 0 3 1 2 0 4 1 0 0 0 2 1 0 0 1 0 0 0 1 0 1 0 0 0 0 1 0 0 1 1 0 2 1 1 1 1 1 1 1 0 0 ? 0 1 4 6 5 0 0 1 0 1 3/4 0/1 0 ? 0 0 0 1 2 1 1 0 ? ? 0 ? ? 2 ? ? ? ? ? ? ?

*Orcinus orca*

0 0 1 0 0 0 2 0/1 3 2 1 0 0 ? 1 0 0 1 0 1 ? 0 0 3 0 0 0 ? 1 0 0 1 1 ? 1 0 4 0 0 1 0 1 1 0 1 0 1 0 1 1 0/1 1 1 ? 1 1 0 0/1 0 ? 0 1 0 1/2 0 1 2 0 0/1 0 1 0/1 0 4 1 2 5 1 0/1 7 1 1/2 0 1 1/2 ? 0 0 0 0 0 1 1 0 2 1 0 2 1 0 1 1 2 ? 1 1 ? ? 0 0/1 0 1 0 2 ? 0 0 0 2 ? 1 ? 3 ? 0 0 0 0 0 0 0 0/1 0 1/3 0 3 0 ? 0 0 1 0 0 2 0 1 2 1 0 0 0 2 1 ? 0 2 1 1 0 2 0 2 2 1 0 0 3 0 2/3 1 0 2 ? 1/2 0 0 ? 3 0 2 0 0 2 0 0/1 0 0/2 3 1 1 0 0 0 2 0 1 2 ? ? 0 0 0 2 0/1 0 1 ? ? 0 0 1 0/1 1 0 0 3 1 2 0 1 2 0/1 1 0 0 1 1 3 2 0 0 2/3 1 0/1 0 1 0 2 0 ? 1 0 0 0 2 1 0 0 1 0 0 1 1 0 1 0 0 1 0 1 1 ? 1 0 1 1 0 1 1 1 1 1 0 0 0 ? 0 1 2 5 4 0 0 1 0 1 2 2/3/4 0 0 0 0 0 1 2 1 1 1 ? ? 1 ? ? 2 ? ? ? ? ? ? ?

*Orcaella brevirostris*

0 0 0 0 1 1 2 0 3 2 1 0 0 ? 0 0 0 1 0 1 0 0 0 3/4 0 ? 0 ? ? ? ? ? 1 ? 0 1 4/5 0 0 1 0 1 1 0 ? 0 1 1 1 1 0 1 1 ? 0/1 1 0 0 0 ? 1 0 0 0/1/2 0 1 2 0 1 0 1 1 0 3 1/2 2 5 1 0 6 0 1/2 0 1 2 ? 0 0 1 1 0 1 1 0 2 1 0 2 1 0 1 1 2 1 1 0 ? ? 0 0/1 0 1 0 2 ? 0 0 0 1 2 1 4 3 1 1 1 0 0 0 0 0 0 0 3 0 2 0 ? 0 0 ? 0 0 1 1 ? 2 0 0 0 0 1/2 2 ? 1 0 1 1 1 2 ? 2 2 1 0 0 3 0 4 1 0 3 1 1 0 2 ? 3 0 2 0 0 2 0 2 0 3 3 0 1 0 0 0 2 0 0 2 ? ? 0 0 0 2 0 0 1 3 ? ? 0 1 1 1 0 0 2 1 2 0 1 2 0 1 0 1 0 0 2 1/2 0 0 3 1/2 0 0 1 1 2 0 3 1 1 0 1 2 2 0 0 1 0 0 0 1 0 1 1 ? 0 0 1 1 0 1 0 0 2 1 1 0 1 1 1 2 0 0 ? ? 1 0 3/4 3/4 0 0 ? 0 ? 4/5 4 ? 0 0 0 0 0/1 2 1 1 1/2 ? ? 0 ? 2 2 ? ? ? ? ? ? ?

*Phocoena phocoena*

0 0 0 0 0 0 2 0 2/3 2 0 0 0 ? 1 0 0 0 0 0 0 0 0 6 0 0 0 0 0 0 0 1 1 ? 2 0 6/7 0 0 1 0 1 1 0 0 0 2 0 2 ? ? 1 1 ? 2 1 2 0 0 ? 0 1 0 0/1/2 0 1 2 2 2 0 1 1 0 1/2 1/2 2 5 0 0 5 1 0 1 0 1 ? 0 0 0 2 0 1 0 0 2 1 0 2 1 0 1 1 2 1 1 0 ? ? 0 0/1 0 ? 0 2 ? 0 2 0 3 1 0/1 4 4 2 0/1 0 0 1 0 0 0 0 0 3 1 2 0 0 0 0 ? 0/1 0 0 1 1 2 0 0 0 ? 1 2 ? 0 2 0 1 0 2 0 2 ? 1 1 0 3 0 4 3 0 2 0 1 1 0 ? 2 0 2 0 0 2 0 1 0/1 0 0 1 1 0 0 0/1 2 0 1 2 0 1 1 0 0 2 1 0 1 3 1 ? 0 1 1 0/1 0 0 3 1 1 0 1 2 0 0 0 1 0 0/1 1 1 0 0 3 4 0 0 1 0 1 0 4 0 0/1 0 1 0 1 1 0 1 0 1 0 1 0 1 0 0 0 0 1 1 0 0 0/1 1 2 0 1 0 1 1 0 2 0 0 0 0 1 4 3 4 0 0 1 0 1 5 5 1 1 0 0 0 1 2 1 1 0 1 1 0 ? 2/3 2 ? ? ? ? ? ? ?

*Phocoenoides dalli*

0 0 0 0 0 0 2 0 3 2 0 0 0 ? 0 0 0 1 0 1 0 0 0 5 0 0 0 ? 0 0 0 1 1 ? 0/1 ? 5/6/7 0 0 1 0 1 1 0 0 0 1 0 4 ? 0 1 1 ? 2 1 0 0 0 ? 1 1 0 1 0 0 2 2 1/2 1 1 0/1 0 2 1 2 5 0 0 5 1 1 1 0 1 ? 0 0 0 0/1 0 1 1 ? 2 1 0 2 1 ? ? ? 1/2 ? 1 ? 0 3 0 0/1 0 ? 0 2 ? 0 2 1 1/2 1/2 1 4 4 2 1 0 0 1 0 0 0 0 0 3 ? 3 0 ? 0 0 0 0/1 0 0 0/1 1/2 2 0 0 1 0 1 2 ? 0 1 0/1 1 0 2 0 2 2 1 1 0 3 0 4 3 0 2 1 3 1 2 ? 2 ? 2 ? 0 2 0 1 0 0/1 1 1 1 0 0 0 2 0 1 2 0 ? 0 1 0 2 1 0 1 3 1 ? 0 1 1 0 0 0 3 1 1 0 1 2 0 0 0 1 0 0 2 1 0 0 3 3 1 0 1 1 2 0 2/3 0 0/1 0 1 1 1 0/1 0 1 0 0 0 1 0 1 1 ? 0 0 2 1 0 0 1 0 2 0 1 1 1 1 1 2 0/1 0 2 0 1 5 1 0 0 0 1 0 0 6/7 6 ? ? 0 0 0 1 2 1 1 0 1 ? 0 ? 2 2 ? ? ? ? ? ? ?

Monodontidae

0 0 1 0 0 ? 2 0 2 2 2 0 0 ? 1 0 0 1 0 1 ? 0 0 2 1 0 0 0 0 0 0 1 1 ? 0 0 3 0 0 1 0 0 1 0 0 0 1 0 1 ? 0 1 1 ? 2 1 1 1 0 ? 1 1 1 2 0 0 2 0 1 0 1 1 0 4 1/2 2 5 0 0 8 1 2 ? ? 2 ? 0 0 1 0 0 1 1 0 2 ? 0 2 1 0 1 1 2 1 1 0 ? 0 1 0 0 ? 0 2 ? 1 2 0 1 2 1 5 1 1 0 1 0 1 0 0 0 0 0 3 1 2 0 0 0 0 ? 0 0 1 1 0 2 0 0 0 1 2 2 ? 0 2 0 1 0 2 0 2 2 1 0 0 0/1 1 0 0 ? 3 0 2/3 0 0 ? 2 ? 2 0 0 2 0 1/2 0 3 2 1 1 0 0 0 2 0 1 2 0 0/1 1 0 0 2 ? 0 1 3 2 ? 0 1 1 1 0 0 1 1 1/2 0 1 2 0/1 1 0 1 1 0 3 1 0 0 3 4 0 0 1/2 2 1 1 3 1 0 0 1 2 1 0 0 1 1 0 0 1 0 1 1 ? 0 ? 1 1 0 1 0 1 2 1 1 0 1 1 1 0 0 0 0 0 0 0 4/5 3 0 0 1 1 1 2/3/4 2/3/4/5/6 1 1 0 0 0 2 ? 1 1 2 0 1 0 ? 1 0 ? ? ? ? ? ? ?

*Inia geoffrensis*

0 0 1 0 0 0 2 0 2 1 3 0 0 ? 1 0 0 1 1 ? ? 0 0 6 0 2 0 0 1 1 0 1 1 ? 1 1 8 0 2 0 0 0 1 0 0 2 3 1 1 0 0/1 1 1 ? 0 1 1 0 0 ? 0 0/1 0 0/1/2 0 0 2 2 1 0 1 2 0 5 1/2 2 5 0 0 7 1 0 0 0 1 ? 0 0 1 0 0 1 0 0 2 1 0 2 1 1 ? 0/1 1 1 1 1 0 ? 0 0 0 0 0 2 ? 0 1 0 1/2 1/2 1 5 1 2 1 0 0 1 0 1 0 0/1 0 1 0 2/3 0 0 0 0 0 0 1 0 1 1 2 0 0 0 1 1 1 0 1 1 2 1 ? 2 1 0 3 1 1 0 3 0 2 2 0 3 0 2 0/1 2 1 3 0 2 0 1 1/2 0 1 0 0/1/2 3 0 1 0 1 3 1/2 0/1 0 2 0 2 0 2 0 1 1 0 1 3 3 ? 0 1 1 1 0 0 2 1 2 0 1 2 0 0 0 1 0 1 1 0 0 0 2 2 0 1 2 0 0/1 0 1 1 1 0 1 0 1 0 0 1 1 1 0 1 0 0 0 1 0 0 2 0/1 0 1 1 0 2 1 1 1 1 1 1 2 0 0 1 1 0 0 3 4 0 1 1 1 1 0 1 1 0 0 0 0 2 ? 1 1 2 ? 1 0 ? 1 1 0 0 0 1 0 1 2

***Isthminia panamensis*** gen. et sp. nov.

? 0 2 0 0 1 2 0 0 0 2 0 0 ? 1 0 0 ? ? ? ? 0 0 3/4 0 1 0 0 1 1 0 1 1 ? 1 1 6 0 2 0 0 0 1 0 ? 2 1 0 1 0 1 1 1 0 0 1 1 ? 0 ? 1 1 0 2 0 0 2 1 1 ? 1 1 0 2 ? 2 5 1 1 5 0 0 0 ? ? ? 0 0 0 0 0 1 0 ? ? ? ? ? ? ? ? ? ? ? ? ? 0 ? 0 0 0 0 0 2 0 1 0 0 2 3 1 5 1 2 1 0 0 0 0 1 0 0 0 2 1 2 0 ? 0 0 0 0 1 1 ? ? ? ? 0 0 ? 2 1 0 0 ? ? ? ? ? ? ? ? ? ? ? ? ? 2 3 0 ? ? ? ? ? ? 3 ? ? ? ? ? ? ? ? ? 3 ? ? ? ? ? ? ? ? ? ? ? ? ? ? ? ? ? ? ? ? ? ? ? ? ? ? ? ? ? ? ? ? ? ? ? ? ? ? ? ? ? ? ? ? ? ? ? ? ? ? ? ? ? ? ? ? ? ? ? ? ? ? ? ? ? ? ? ? ? ? ? ? ? ? ? ? ? ? ? ? ? ? ? ? ? ? ? ? ? ? ? ? ? ? ? ? ? ? ? ? ? ? ? 0 0 ? ? ? ? ? ? ? ? ? ? ? 1 1 2 0 0 ? 1

*Pontoporia blainvillei*

0 0 2 0 0 1 2 0 2 1 1 0 0 ? 1 0 0 1 0 1 ? 0 0 8 0 0 0 0 0 0 0 1 1 ? 0 1 9 0 2 0 1 0 1 0 0 0 3 0 1 1 1 1 1 0 1 1 1 0 0 ? 1 1 0 1 0 0/1 2 2 1 0 1 2 0 4 1 2 5 1 2 7 1 0 0 0 1 ? 0 0 1 0 0 1 0 0 2 1 0 2 1 1 1 0/1 1 1 1 1 0 ? 0 0 ? 1 0 1 0 0 0 0 1 2/3/4 1 5 2 1 2 0 0 1 1 0 0 0 0 1/3 1 1 0 0 0 0 0 0 0 0 1 0 1 0 0 1 0 2 1 0 0 0 0 1 0 2 1 2 2 0 0 0 1 0 2 3 0 2 0 2 1 2 1 3 ? 2 1 0 2 1 0 0 0 3 0 1 0 0 1 1 0/1 1 2 0 2 1 1 0 1 1 0 1 3 3 ? 0 1 1 1 0 0 3 1 2 0 0 2 0 0 1 1 0 1 2 2 0 0 3 4 1 0 1 0 2 0 1 1 0/1 0 1 0 2 1 1 1 0 0 0 1 0 1 0 1 0 0 2 1 0 0 0 1 2 0 0/1 0 1 1 0 0 0 0 0 1 0 0 6 6 0 1 1 1 1 2 0 1 0 0 ? ? 2 ? 1 1 2 ? 0 0 ? 3/4 2 0 1 2 0 1 0 0

*Lipotes vexillifer*

0 0 1 0 0 1 1 0 2 0 2 0 0 ? 1 0 0 1 0 0 ? 0 0 7 0 2 0 0 0 0 0 1 1 ? 1 1 8 0 2 0 0 0 1 1 0 2 4 0 1 1 0 1 1 0 1 1 2 0 0 ? 0 1 0 0 0 0 2 0 2 0 1 2 0 4 1 2 5 1 1 7 0 2 0 0 0 ? 0 0 0 0 0 1 0 2 2 1 ? 2 1 1 ? ? 0/1 ? 1 0 0 ? 0 0 0 1 0 2 ? 1 1 0 1 2 1 4 3 2 2 0 0 0 1 1 0 1 0 2 1 3 0 ? 0 0 0 0 2 0 1 1 2 0 0 0 0 2 1 ? 1 1 2 1 0 2 0 2 3 1 1 0 3 1 3 3 0 0/1 ? 1 1 2 1 3 0 2 0 0 2 0 0 0 1 3 1 1 0 0 1 2 1 1 2 ? ? 1 2 1 2 1 0 1 1 2 3 0 1 1 1 0 0 0 1 2 0 0 2 1 1 2 0 0 0 2 1 0 0 3 4 1 0 1 0 2 0 2 1 0 0 1 0 1 0 0 0 0 0 1 1 0 1 1 0 1 0 1 0 0 1 1 0 2 0 0 1 1 1 ? 0 0 0 2 0 0 0 5/6 5 0 0 1 0 1 2/3 1/2 0 0 0 0 0 1 2 1 0 2 ? 0 0 ? 4 1 1 0 0 1 1 0 ?

*Mesoplodon*

0 0 ? 1 1 0 2 0 3 0 2 0 0 ? 1 0 0 2 0 1 0 ? ? 0 ? 0 2 ? ? ? ? ? ? ? 1 ? 1 0 0 1 0 1 1 0 1 0 1 0 1 1 0/1 1 0 0 1 1 1 2 0 ? 1 1 0 0 1 0 2 0 1 0 1 1 0 5 1 2 5 1 1 7 1 0 0 0 2 1 0 0 0 2 2 0 0 0 2 1 0 2 ? ? 1 ? ? 1 ? ? 1 6 0 2 ? 0 0 2 2 0 0 1 1 2 1 5 4 1 1/2 1 0 1 0 0 0 0 0 3 ? 3 ? ? 0 0 0 0 1 1 1 0 1/2 0 0 0 1 0 2 ? 0/1 1 0 1 1 1 ? 2 3 1 0 0 0/1 0 0/1/2 0/1/2 ? 2 0 4 0 1 1 3 ? 1 0 1 2 0 1 0 3 0 1 1 0 0 0 2/3 0 1 2 1 ? 0 1 0 2 0 0 1 2/3 2 3 0 1 ? 1 0 0 3 1 1 0 1 1/2 1 0 2 1 0/1 1 1 1 0 0 3 4 1 0 2 1 2 1 ? 0 0 0 1 2 1 0 0 1 0 1 0 1 1 ? 0 1 ? 0 0 0 0 1 1 2 2 1 0 0 ? ? ? 0 0 1 2 0 1 1 6 4 1 1 1 0 2 5 2 0 0 0 0 0 0 0 1 2 0 0 0 1 0 2/3 2 ? ? ? ? ? ? ?

*Tasmacetus shepherdi*

0 0 1 0 1 1 2 0 3 0 1 0 0 ? 0 0 2 1 0 ? ? 0 0 5 0 0 0 1 0 0 0 1 1 ? ? 2 7 0 1 0 0 0 1 0 0 ? ? 0 2 1 1/2 1 0 1 1 1 1 ? 0 ? 1 1 0 0 0 0 2 0 1 0 1 1 0 5 1 2 5 0 0 7 1 1 0 0 1 1 0 0 0 2 ? 1 0 0 2 ? 0 2 0 ? 1 0/1 ? 1 1 1 1 7 0 1 0 1 0 1 2 1 0 0 2 2 1 5 4 0 2 1 ? 0 ? 0 0 0 0 2/3 ? 3 ? ? 0 0 0 1 0 1 1 1 2 0 0 0 2 0 0 ? 0 0 0/1 1 0 1 ? 2 3 1 0 0 1 0 0/1/2 0/1/2 ? 2 0 3 0 1 1 3 ? 0 0 0 2 0 1 1 3 1 1 1 0 0 0 4 ? 1 1 ? ? 1 0 0 2 0 0 0 2 2 3 0 1 1 2 0 0 3 1 0 1 1 2 1 0 1 1 1 2 1 1 0 0 3 2 1 0 ? 2 1 0 2 0 1 0 1 2 1 0 0 1 0 1 0 1 0 0 0 1 1 0 1 0 0 1 1 2 2 0 0 1 0 0 0 1 0 0 3 0 1 3 6 3 1 0 1 0 1 5 1 0 0 0 0 0 1 0 1 2 0 ? 0 1 0 2/3 2 ? ? ? ? ? ? ?

*Ziphius cavirostris*

0 0 1 0 0/1 0 2 0 3/4 2 1 0 0 ? 0/1 0 0 2 0 1 1 ? 0 0 ? ? 0 1 ? ? ? ? ? ? 0 ? 1 0 0 1 0 0 1 0 0 0 1 0 1/2/3 1 1 1 0 0 2 1 1 2 0 ? 0 0 0 0/1 0 0 2 0 1 0 1 0 0 5 1 2 5 1 1 5 0 0 0 0/1 3 1 0 0 0 2 1 0 0 0 2 0 0 2 0 ? 1 0 0 1 1 0 0 4 0 1 0 2 0 2 0 1 0 0 2 1 0 4 4 1 1 0 0 1 0 0 0 0 0 3 0 2 ? ? 1 0 0 1 1 1 1 0 2 0 0 0 0 0 0 ? 0 0/1 0 1 0 0 ? 2 3 1 0 0 0 0 0 0 ? 2 0 3 0 1 ? ? 0 1 0 0 2 0 1 ? 3 2 0 1 0 1 0 3 0/1 1 2 ? ? 1 0 1 3 0 0 0 1 2 3 0 1 1 ? 0 0 3 1 2 0 1 2 0 0 3 1 0 2 1 3 0 0 3 2 1 1 3 2 2 0 1 1 1 0 1 2 1 0 0 1 0 0 0 1 0 0 0 1 0 0 1 1 0 1 1 2 0 ? 0 1 0 0 1 0 1 0 2 0 1 2 6 4 1 0 1 0 1 4/5 1/2 0 0 0 0 0 1 1 1 2 0 0 0 1 0 2 2 ? ? ? ? ? ? ?

*Berardius*

0 0 1 0 1 0 2 0 3 2 2 0 0 ? 0 0 2 2 0 1 0 ? ? 0 ? 0 2 1 ? ? ? ? ? ? 1 2 2 0 0 1 0 0 1 0 0 0 1 0 1 1 1/2 1 0 0 3 0 1 2 0 ? 1 1 0 0/1/2 1 0 2 0 1 0 1 1 0 5 2 2 5 1 0 6 0 0 0 0 2 1 0 0 0 1 0 1 0 1 2 1 0 2 0 ? 1 1 1 1 1 ? 1 6 0 1 ? 0 0 2 1 1 0 0 2 1 0 5 3 1 2 1 ? 0 0 0 0 0 0 3 ? 3 ? ? 0 0 1 1 0 0 1 0 2 0 0 1 1 0 0 ? 0 0 0 1 1 1 ? 2 3 1 0 0 0 0 0 0 ? 2 0 4 0 1 1 3 ? 0 0 0 2 0 0 ? 3 1 1 1 0 0 1 3 0 1 2 1 2 0 1/2 0 4 0 0 1 ? 2 3 0 1 1 1 0 0 3 1 2 0 1 2 1 0 3 0 0 1 1 1 0 0 3 4 1 1 3 2 2 0 ? 0 0 0 0 1 1 0 0 ? 0 1 0 1 1 1 0 1 1 0 1 1 ? ? 1 2 2 1 0 1 1 1 0 2 0 0 ? 0 1 1 5 3 1 0 0 0 ? 5 1 0 0 0 0 0 1 1 0 1 0 0 ? 1/2 0 3 2 ? ? ? ? ? ? ?

*Platanista*

0 0 1 0 0 1 3 0 1 2 0 0 0 ? 0 0 0 1 ? ? 0 1 0 6/7 ? 0 0 1 ? ? ? ? 1 ? 1 1 7 0 2 0 1 0 1 0 0 2 4 1 1 ? ? ? ? ? ? 0 3 1 0 ? 1 0 0 2 0 0/1 2 0 3 1 1 1 0 4 1/2 2 5 1 3 7 0 0 0 1 1 ? 0 1 0 1 0 1 0 3 2 1 0 2 0 ? 1 0/1 2 1 1 0 0 ? 0 0 0 1 0 2 ? 0 1 0 0 3 ? 4 1/2 2 1 1 0 0/1 0 1 0 0 0 1 1 1 0 0 0 0 0 0 0 0 1 0 2 0 0 1 1 1 1 0 0 0 0 1 2 ? ? ? 2 0 0 1 1 0 0 0 ? ? ? 3 0 0 0 3 0 1 1 0 2 ? ? ? 0 4 0 1 0 0 0 1/2 1 0 2 0 ? 1 2 0 2 1 0 1 0 ? 3 0 1 1 1 0 0 0 1 2 0 1 2 0 0 0 1 0 0 2 ? 0 1 ? 3 1 0 3 2 2 3 0 1 0 0 1 0 2 0 0 1 2 ? 0 1 1 0 0 1 0 ? ? 0 0 ? 1 0 2 ? 0 0 0 ? ? 0 0 ? ? 0 0 0 5/6 4 0 ? ? ? ? 3 3/4 0 0 0 1 1 ? ? 1 0 2 0 1 0 ? 4 1 ? ? ? ? ? ? ?

*Physeter macrocephalus*

0 0 1 1 0 0 1 0 3 2 2 0 0 ? 0 0 2 1 0 1 1 ? ? 1/2/3 ? ? 0 0 0 ? 0 1 ? ? 0/1 0/1 6/7 0 2 1 0 0 1 ? 0 0 1 1 1 ? 1 1 1 0 1 0 1 0 0 ? 1 0 0 0 0 0 2 0 1 2 1 0 0 5 0 2 5 1 1 7 1 0 0 2 3 0 1 1 0 0 0 1 0 3 1 0 1 2 1 1 0 0 0 1 0 0 ? ? 0 0 ? 1 1 ? ? ? 0 0 ? ? 1 5 0/1 2 ? 0 0 1 1 ? 1 ? 0 1 0 3 ? ? 0 0 0 1 0 2 0 ? 2 1 0 1 0 2 0 ? 0 0 0 ? 0 0 ? 2 2 1 0 0 3 0 0 0 ? ? ? 3 1 1 ? 3 0 1 0 0 2 0 1 0 3 1 1 0 0 0 ? 3 0 ? 2 1 0 0 2 0 2 1 0 1 3 2 3 0 1 1 1 0 0 2 1 1 0 1 2 1 0 0 1 0 1 2 2 0 0 3 1 0 0 1/2 1 ? 0 2 1 2 0 0 1 1 0 0 1 0 1 1 1 1 0 0 0 1 0 ? 1 ? 1 1 2 2 1 0 0 1 1 0 1/2 0 1 3 0 0 5 5 2 1 ? ? ? 0 3 3 0 0 0 0 1 1 1 1 0 0 0 1 2 ? 0 1 ? ? ? ? ? ? ?

*Kogia*

0 0 1 1 0 0 2 0 4 2 3 0 0 ? 0 0 0 1 0 0 0 ? 0 0 ? 0 0 0 0 ? ? ? ? ? 1 0 5 0 0 1 0 0 1 0 0 0 0 1 0 0 0/1 1 1 1 1 1 1 0 0 ? 0 1 0 1/2 0 0 2 0 1 2 1 0 0 4 ? 2 5 1 2 4 1 0 0 2 3 0 1 1 0 0 0 0 0 1 1 0 1 2 1 1 0 0 0 1 0 0 ? ? 0 1 0 0 2 2 ? ? ? ? ? ? ? ? ? ? ? ? 1 2 1 ? 1 ? 0 1 ? 3 0 ? 0 0 ? 0 0 2 0 ? 2 1 0 0/1 0 2 0 ? 0 0 0 ? 1 0 ? 2 3 1 0 0 1 1 0 0 ? 3 ? 0 ? ? ? ? 0 0 0 0 2 0 0 0 3 2 1 1 0 0 1 3 0 1 2 1 0 1 0 0 ? 1 0 0 3 2/3 ? 0 1 ? 2 0 1 3 1 2 0 1 2 0 0 0 1 0 2 1 1 0 1 3 2 1 0 0 1 1 0 4 0 0 0 1 1 1 1 1 1 0 1 0 1 1 0 1 ? ? 0 2 1 0 1 1 2 1 1 0 0 0 1 0 0 0 0 0 1 1 5 3/4 4 0 0 1 0 1 4/5 2 0 0 0 0 0 0 1 1 1 0 ? 1 0 ? 2 2 ? ? ? ? ? ? ?

*Zygorhiza kochii*

? 0 0 0 0 0 2 0 0/1 2 0 0 0 0 0 0 0 0 0 0 ? 0 4 1 0 1 ? ? 0 2 1 0 0 0 0 1 4 0 1 1 0 0 1 1 0 0 2 ? 5 1 0 0 0 0 1 0 0 ? 2 2 0 1 0 1/2 0 ? 2 1 0 ? ? ? ? 0 ? 0 1 ? ? 2 0 0 0 0 1 ? ? 0 0 0 0 0 ? ? ? ? ? ? ? ? ? ? ? ? ? ? 0 1 0 0 ? 0 0 1 0 0 0 0 3 1 1 0 0 2 ? 0 ? 2 0 ? 1 ? 0 0 ? 0 1 2 0 ? ? ? 0 2 1 2 2 0 0 0 1 1 1 0 0 1 0/1 0 0 0 ? ? 2 ? 0 ? 2/3 0 0/1/2 0/1/2 ? ? ? ? ? 0 ? 1 ? ? ? 0 ? ? ? ? ? 1 0 1 0 0 0 2 0 1 2 0 0 0 0 0 2 0 0 0 ? 0 0/1 0 1 0 ? 0 0 ? 1 1 1 0 0 0 ? ? 1 0 ? ? ? 0 0 0 0 0 ? ? ? ? 0 ? 1 1 0 1 2 1 0 0 0 ? 1 0 ? ? 1 0 ? ? ? ? ? 0 ? ? ? 2 0 0 0 0 1 ? 0 0 0 0 1 0 0 1 1 0 0 0 0 1 6 ? ? 0 0 0 0 0 2 0 0 0 ? ? ? ? ? ? ? ? ? ? ? ? ?

*Archaeodelphis patrius*

? ? ? ? ? ? 2 ? ? ? 0 0 0 ? 1 0 ? 0 0 1 ? ? ? ? ? ? ? ? ? ? ? ? ? ? ? ? ? ? ? ? ? ? ? ? ? 0 3 0 2 1 1 1 0 0 0 ? 2 1 1 2 ? 0 0 ? 1 1/2 0 0 ? ? ? ? 0 1 0 1 2 0 0 3 0 0 0 0 1 ? 0 0 0 2 0 0 0 ? ? ? ? ? ? ? ? ? ? ? ? ? 0 2 0 0 ? 1 0 1 0 0 0 0 2 1 1 2 1 1 ? 0 0 ? 0 0 1 ? 0 0 ? 0/1 0/1 1/2 0/1/2 0 ? ? ? 2 0/1 1 1 0 0 ? 0 ? ? ? ? ? 0 0 0 0 ? 1 2 0 0 ? ? 0 0 0 ? 4 ? 1/2 0 0 ? 1 ? 1 0 0 2 ? ? ? 0 ? 0 1 0 1 0 1 0 ? 1 ? ? 0 0 1 3 0 ? ? 0 1 0/1 0 2 1 ? 0 0 ? 1 1 0 0 2 0 ? ? 0 1 ? 1/2/3 ? 0 ? ? ? ? ? ? ? ? ? 0/1 ? ? ? 1 0/1 ? ? ? 1 ? ? ? ? ? 1 ? ? ? ? ? ? 0 1 0 1 ? ? ? ? ? ? ? ? ? ? ? ? ? ? ? ? ? ? ? ? ? ? ? ? ? ? ? ? ? ? ? ? ? ? ? ? ? ? ? ? ? ? ? ? ? ?

*Xenorophus* sp.

? 0 1 0 0 0 2 0 2 2 1 0 0 ? 1 0 ? 0 0 1 ? 0 3 2 1 1 ? ? 0 1 0 1 1 ? ? ? ? ? ? ? ? ? ? ? ? 0 2 0 2 1 2 1 0 0 1 ? 1/2/3 1 0 ? 1 0 0 2 1 0 1 0 1/2/3 ? 0 0 1 3 0 2 4 ? ? 4 1 0 0 0 0 ? 0 0 0 1 1 0 0 ? ? ? ? ? ? ? ? ? ? ? ? ? 0 2 0 0 0 2 0 1 0 0 0 0 1 2 1 2 1 1 1 0 0 2 0 0 0 1 0 0 1 0 1 1 1 0 1 0 0 2 2 3 2 0 0 0 0 0 1 0 0 1 0/1 0 0 ? ? 0 2 ? 0 0 ? 0 0/1/2 0/1/2 ? 2 1 ? 0 ? ? 1 ? 0 ? 0 1/2 1 2 1 1 0 0 1 0 1 0 4 ? 0 2 ? ? 0 0 0 3 0 0 1 ? 1 0/1 0 2 1 1 0 0 2 1 1 0 0 2 ? ? 1 ? ? ? ? ? 0 1 2 2 ? 1 0/1 0 1 0 0 1 1 0 1 0 1 0 0 1 0 1 0 ? ? 1 0 0 ? 0 ? 0 0 1 0 1 2 0 0 0 0 0 0 0 0 0 ? ? ? ? ? ? ? ? ? ? ? ? ? ? ? ? ? ? ? ? ? ? ? ? ? ? ? ? ? ? ? ? ? ? ? ?

*Xenorophus sloanii*

? ? ? 0 0 0 2 ? 3 2 1 0 0 ? 1 0 0 0 0 1 ? 0 4 2/3/4 1 2 ? ? 0 1 1 0 0 1 ? ? ? ? ? ? ? ? ? ? ? 0 2 0 2 1 2 1 0 0 1 ? ? 1 0 ? ? 0 0 2 1 ? 0 0 ? ? ? 0 1 3/4/5 0 2 3/4/5 0 0 4 1 0 0 0 1 ? 0 0 0 1 1 0 ? ? ? ? ? ? ? ? ? ? ? ? ? ? 0 2 0 0 ? 2 0 1 0 0 0 0 2 2 0 2 1 1 1 0 0 ? 0 0 0 1 ? ? ? ? 1 ? ? ? ? ? ? ? ? ? ? ? ? ? ? ? ? ? ? ? 0/1 0 0 0 ? 0 2 ? 0 ? ? ? 0/1/2 0/1/2 ? ? ? ? ? ? ? ? ? ? ? ? ? ? ? ? ? ? ? ? ? ? ? ? ? ? ? ? ? ? ? ? ? ? ? ? ? ? ? ? ? ? ? ? ? ? ? ? ? ? ? ? ? ? ? ? ? ? ? ? ? ? ? ? ? ? ? ? ? ? ? ? ? ? ? ? ? ? ? ? ? ? ? ? ? ? ? ? ? ? ? ? ? ? ? ? ? ? ? ? ? ? ? ? ? ? ? ? ? ? ? ? ? ? ? ? ? ? ? ? ? ? ? ? ? ? ? ? ? ? ? ? ? ? ? ? ? ? ? ? ?

*Agorophius pygmaeus*

? ? ? 0 0 0 1 ? 4 2 2 0 0 ? 1 0 0 1 0 ? ? 0 4/5/6 ? 0 ? ? ? ? ? ? ? ? ? 0 ? 4 0 ? 1 0 0 ? ? ? 0 2 0 3 ? ? ? 0 1 1 ? 1 0 0 ? 1 0 0 2 0 1 0 0 1 0 0 1 0 3 1 2 3 0 0 3/4 0 ? ? 0 1 ? 0 0 0 0 0 ? 0 ? ? ? ? ? ? ? ? ? ? ? ? ? 1 4 1/2 0 1 ? 0 1 0 ? 0 0 3 1 1 2 1 1 0 0 0 1 0 0 0 0 1 0 1 2 1 2 1 ? 1 0 0 0 1 0 2 0 0 0 ? 0 1 0 1 1 0/1 ? ? ? ? ? 2 ? 0 ? ? ? 0/1/2 0/1/2 ? ? ? ? 0 ? ? 0 ? 1 ? ? 1/2 0 1 1 ? ? 0 ? ? ? ? ? ? ? ? ? ? ? ? ? ? ? ? ? ? ? ? ? ? ? ? ? ? ? ? ? ? ? ? ? ? ? ? ? ? ? ? ? ? ? ? ? ? ? ? ? ? ? ? ? ? ? ? ? ? ? ? ? ? ? ? ? ? ? ? ? ? ? ? ? ? ? ? ? ? ? ? ? ? ? ? ? ? ? ? 0 0 ? ? ? ? 0 0 1 3/4/5/6/7 ? ? ? ? ? ? ? ? ? ? ? ? ? ? ? ? ? ? ? ? ? ? ? ?

*Patriocetus kazakhstanicus*

? 0 1/2 0 0 0 2 ? 4 2 1 0 0 ? 1 0 0 2 0 0 ? 0 6 3 0 1 ? ? 0 1 1 0 0 0 ? ? 3/4/5/6/7/8/9 0 ? ? 0 ? ? ? ? 0 ? 0 ? ? 0 ? 0 1 0 ? 1 ? 0 ? 0 1 0 ? 0 1 0 0 1 0 0 1 0 3 1 2 4 0 0 5 0 0 0 0 1 ? ? 0 0 0 0 ? ? ? ? ? ? ? ? ? ? ? ? ? ? ? 1 4 1 0 ? 0/1 0 1 ? ? 0 0 5 0 0 2 2 1 0 0 0 1 0 0 0 0 0 0 ? 2 ? ? 0/1 ? ? 0 0 ? 1 1 1 0 0 0 1 1 1 0 1 ? 0/1 ? 0 ? ? ? 1/2 ? ? ? ? ? 0/1/2 0/1/2 ? ? ? ? 2 ? ? 3 ? ? 0 0 1/2 1 1 ? 0 3 0 1 0 0 1 3 ? 0 2 ? ? ? ? ? ? ? ? ? 0/1 ? ? 0 2 ? ? 0 ? ? ? ? ? ? ? ? ? ? ? ? ? ? ? ? ? ? ? ? ? ? ? ? ? ? 0 1 1 1 1 1 0 0 1 ? ? ? ? ? ? ? ? ? ? ? ? ? ? ? ? ? ? ? ? ? ? ? ? ? ? ? ? ? ? ? ? ? ? ? ? ? ? ? ? ? ? ? ? ? ? ? ? ? ? ? ? ? ? ? ? ? ? ? ? ? ?

*Waipatia maerewhenua*

? ? 1 0 0 0 2 ? 4 2 1 0 0 ? 1 0 ? 0 0 0 ? 0 3/4/5 4 1 2 0 ? 0 1 0 0 0 0 0 1 6/7/8/9 0 1/2 1 0 0 1 0 1 0 2 0 2 1 1 1 1 0/1 0 ? 1/3 ? 0 ? 1 0 0 ? 0 1 1 0 1 0 0 1 0 4 2 2 4 0 0 5 0 0 0 0 0 ? 0 0 0 0 0 0 0 ? ? ? ? ? ? ? ? ? ? ? ? ? 1 4 1 0/1 ? 0/1 0 1 0/1 0 0 0 1 1 0 3 2 1 1 0 ? 1 0 0 0 0 ? 1 ? 2 ? ? 1 0 1 0 0 1 1 1 1 0 0 0 1 1 0 ? 0 1 0/1 1 0 0/1 ? ? 2 ? 0 ? ? ? 0/1/2 0/1/2 ? ? ? ? 1 0 1 2/3 ? 1/2 0 0 2 ? 2 1 1/3 3 0 ? 0 0 0 2 0 0 2 ? ? 0 1 1 3 1 1 1 2 2 3 0 1 1 1 0 0 2 1 1 1 ? 2 0 ? ? 1 0 1 1 1 0 0 2 3 0 0 0 0 0 3 0 ? ? 0 1 2 1 0 0 1 0 1 0 ? ? 1 0 0 0 ? ? 0 0 ? 0 0 2 1 0 0 0 ? ? ? 0 ? ? ? 0 ? ? ? ? ? ? ? ? ? ? ? ? ? ? ? ? ? ? ? ? ? ? ? ? ? ? 0 0 0 0 1 0 0

*Squalodon calvertensis*

? 1 1 0 0 0 2 ? 4 3 1 0 0 ? 1 0 0 2 0 1 ? 0 5 3 1 1 ? 0 0 1 0 0 0 0 ? ? 4 0 1 1 0 0 1 0 0 0 2 0 2 1 0 1 0 0/1 1 ? 1 ? 0 ? 0 1 0 2 0 1 1 0 1 0 1 0 0 4 2 2 4/5 0 0 7 0 1 0 0 1 ? 0 0 0 0 0 0 0 ? ? ? ? ? ? ? ? ? ? ? ? ? 0 6 2 0 ? 0 0 1 0 0 0 0 2 1 0 4 0 1 1 0 0 1 1 0 0 0 0 2 ? 3 0 1 1 0 1 0 0 2 1 1 2 0 0 0 0 2 0 1 0 0 0/1 1 ? 0/1 ? ? 2 ? 0 ? 1 1/2 0/1/2 0/1/2 ? ? ? ? 0 0 1 2 ? 2 0/1 0 1/2 1 0 0 3 3 0 1 0 0 0 2 1 0/1 2 ? ? 0 1 1 2 1 1 1 2 2 0/1 0 1 1 1 0 0 0 1 0 0 0 2 1 0 0 1 0 0 2 1 0 0 3 4 1 0 2 0 1 1 2 0 1 0 1 0 1 0 0 1 ? ? 0 ? ? 1 ? ? ? ? ? ? ? ? ? ? 2 1 0 0 0 0 0 2 0 0 ? 1 0 0 ? ? ? ? 1 0 1 ? ? ? ? ? ? 1 ? ? 1 0 1 1 ? ? ? ? ? ? ? ? ? ? ? ?

*Notocetus vanbenedeni*

? ? 1 0 0 0 2 ? 4 2 1 0 0 ? 0 0 0 2 0 0 ? 0 0/1 4 0 1 ? ? 0 0 0 0 0 0 ? ? 4/5/6/7/8/9 0 2 1 ? 0 1 ? ? 0 2 0 3 ? 0/1 ? ? ? ? ? 1 ? 0 ? 1 0 0 0/1 0 2 ? 0 1 0 1 2 0 4 2 2 5 1 0 7 0 0 0 0 1 ? 0 0 0 0 0 1 0 ? ? ? ? ? ? ? ? ? ? ? ? ? 0 6 0 0/1 ? 1 0 2 ? ? ? ? ? ? 1 4/5 1 ? 1 ? ? 0/1 1 0 0 1 0 2 ? 3 ? 0/1 0/1 1 0 0 0 1 1 0 2 0 0 0 0 2 1 1 0 1 0/1 1 0/1 1 ? 2 3 0 0 ? 2/3 0 3 0/1/2 ? ? ? ? 0 ? ? 3 ? ? 0 0 3 0 2 0 0 3 0 1 0 0 0 1 1 1 2 0 0 1 1 0 3 0 1 1 2 2 3 0 1 1 1 0 0 1 1 1 1 1 1 0 0 0 1 0 2 1 0 0 0 3 4 1 0 3 2 1 2 2 0 1 0 1 1 1 0 0 1 2 0 0 ? ? 1 1 ? 0 0 2 0 0 1 0 0 2 1 0 0 0 0 0 2 0 0 ? 2 0 0 ? 0/1/2/3/4/5/6 ? ? ? ? ? ? ? ? 0 ? 1 1 ? ? ? ? ? ? ? ? ? ? ? ? ? ? ? ? ? ?

*Prosqualodon davidis*

? 0 0 0 0 0 2 1 4 2 2 0 0 ? 0 0 0 1 0 0 ? 0 4 3 0 2 0 0 0 0 0 1 0 0 ? 1 5 0 ? 1 0 0 1 ? 1 0 1 0/1 0 0 0 ? 0 1 0 1 1 0 0 ? 1 1 0 1/2 0 0 0 0 1 0 0 0 0 3 1/2 2 5 1 1 5 0 0 0 0 1 ? 0 0 0 1 0 0 0 ? ? ? ? ? ? ? ? ? ? ? ? ? 1 5 0 0 ? 0 0 1 0 0 0 0 2 1 1 4 2 1 1 0 ? 1 1 0 0 0 ? 2 ? 2 ? ? 0 0 ? 0 0 0 1 0 3 1 0 0 1 1 0 ? 0 ? 0/1 ? 0 2 0 1 2 1 0 0 2/3 1 0/1/2 0/1/2 ? 1 0 3 1 0 1 2/3 ? 2 0 0 2 ? ? ? ? 3 0 1 0 0 0 2 ? 0 1 ? ? 1 ? ? 3 ? ? ? 1/2/3 ? ? 0 1 1 ? 0 0 ? 1 1 ? ? ? ? ? ? 0 1 ? ? ? ? ? ? ? ? ? ? ? ? ? ? 0 0/1 ? ? 2 0 ? ? 1 0/1 0 0 1 0 0 0 0 ? ? ? ? ? ? ? ? 2 0 0 0 ? ? ? 0 0 ? ? ? ? ? ? ? ? ? ? ? ? ? ? 0 ? 0 1 0 ? 1 1 0 0 1 ? ? ? ? ? ? ? ? ? ? ? ?

*Squaloziphius emlongi*

? ? ? ? 0 ? 2 ? 4 2 0/1 0 0 ? 1 0 0 1 0 1 ? ? ? ? ? ? ? ? ? ? ? ? ? ? ? ? ? ? ? ? ? ? ? ? ? 0 1 0 2 ? ? 1 0 ? ? ? 1 ? 0 ? 1 1 0/1 2 0 0 1 0 1 0 1 2 0 4 1 2 5 0 0 6/7 0 0 0 0 1 ? 0 0 0 2 0 0 0 ? ? ? ? ? ? ? ? ? ? ? ? ? 0 5/6 0 1 ? 0/1 0 1 0 0 0 0 2 2 0 4 2 1 1 0 ? 1 1 0 0 0 ? 2 ? 3 ? ? 0/1 0 ? 0 0 2 0 1 2 0 0 0 2 2 0 ? 0 1 ? 1 1 1/2 0 2 3 1 0 0 ? 0 0/1/2 0/1/2 ? 1 0 4 1 0 1 3 ? 1/2 0 1/2 2 0 0 0 3 1 1 1 0 1 0 2 0 1 1 ? ? ? ? ? ? ? ? ? ? ? ? ? ? ? ? ? ? ? ? ? ? ? ? ? ? ? ? ? ? ? ? ? ? ? ? ? ? ? ? ? ? ? ? ? ? ? ? ? ? ? ? ? ? ? ? ? ? ? ? ? ? ? ? ? ? ? ? ? ? ? ? ? ? ? ? ? ? ? ? ? ? ? ? ? ? ? ? ? ? ? ? ? ? ? ? ? ? ? ? ? ? ? ? ? ? ? ? ? ? ? ? ? ?

*Xiphiacetus bossi*

? 0 2 0 0 0 2 0 0 3 0 0 0 ? 1 0 0 2 0 0 ? 0 0 8 0 0 ? ? 0 0 0 1 1 ? ? ? 9 0 2 0 0 0 1 ? 0 0 2 0 2/3 1 0/1 1 1 0/1 ? 1 1 0 0 ? 1 0 0 2 0 1 2 0 1 0 1 2 0 5 1/2 2 5 1 0 7 0 0 0 0 1 ? 0 0 0 1 0 1 0 ? ? ? ? ? ? ? ? ? ? ? ? ? 1 6 0 0 ? 1 0 1 2 1 0 0 4 0 1 5 2 1 1 0 0 1 1 0 0 0/1 0 2 1 3 0 0 0 0 ? 1 0 2 2 2 2 0 0 0 2 2 1 ? 0 1/2 0/1 1 0 0 ? 1 2 0 0 0 1 0 0/1/2 0/1/2 ? ? ? ? 0 0 ? 3 0 2 0 1 2 0 0 0 3 1 1 1 0 0 0 1 0 1 2 0 ? 0 1 0 2 0 1 0 2/3 2 3 0 1 1 1 0 0 2 1 2 0 0 2 0 0 1 1 0 2 1 1 0 0 3 4 0 0 3 0 1 1 1/2 0 1 0 1 1 1 0 0 1 1/2 0 0 ? ? 1 0 1 0 0 1/2 0 0 1 1 0 2 ? 0 0 0 0 0 0 0 ? ? ? 0 0 ? ? ? 0 1 0 1 2/3/4/5/6/7 ? ? ? ? 0 0 1 2 1 ? ? ? ? ? ? ? ? ? ? ? ? ? ? ?

*Zarhachis flagellator*

? 0 2 0 0 0 2 0 0 2 0 ? 0 ? ? 0 0 1 0 ? ? 0 ? 8 ? 0 0 0 ? 0 0 0 0 ? 0/1 1 9 0 2 0 1 0 1 ? 0 0 3 0 1/2 1 ? ? ? ? ? ? 1 ? 0 ? 1 0 0 2 0 1 1 0 1 0 0 2 0 5 1 2 5 1 2 7 0 0 0 0 1 ? 0 0 0 1 0 1 0 ? ? ? ? ? ? ? ? ? ? ? ? ? 0 6 0 0 0 1 0 1 0 0 0 0 1 1 0 5 2 1 1/2 0 0 1 1 0 0 1 0 2 1 3 0 0 0 0 0 0 0 2 1 1 3 1 0 1 0 2 1 ? 0 1 0/1 1 1 2 0 0 3 0 0 0 2/3 0 ? 0/1/2 ? 0 0 1 0 0 0 3 ? ? 0 0 2 1 2 1 0 1 0 1 0 0 0 1 0 1 2 ? ? 0/1 0/1 0 3 0 1 0 0 2 3 0 1 1 1 0 0 1 1 2 0/1 0 2 0 0 0 0/1 0 0/1 1 1 0 1 2/3 4 0 0 2/3 0 1/2 3 0 0 0/1 0 1 1 1 0 0 0 2 1 0 1 0 1 0 0/1 0 0 1/2 ? 0 1 1 1 2 1 0 0 0 1 0 0 0 0 ? 1 0 0 6 6 0 0 1 0 2 2/3/4/5/6/7 ? ? 0 ? ? ? ? ? ? ? ? ? ? ? ? ? ? ? ? ? ? ? ? ?

*Parapontoporia wilsoni*

? 0 2 0 0 1 2 0 1 3 1 0 0 ? ? 0 0 1 0 0 ? 1 0 8 0 ? ? ? ? ? ? ? ? ? ? ? ? ? ? ? ? ? ? ? ? 0 3 0 2 1 0 1 1 ? 0/1 1 ? ? 0 ? 1 0 0 0 0 0 2 0 0 0 1 2 0 4 1 2 5 1 1 7 0 0 0 0 2 ? 0 0 ? 0/1 0 1 ? ? ? ? ? ? ? ? ? ? ? ? ? ? ? ? 0 0 ? 1 0 2 ? 0 1 ? 2 2 1 5 2/3 2 2 1 0 0 1 0 0 0 ? 2 ? 2/3 0 ? 0 ? ? 0 0 1 1 1/2 2 0 0 2 1 2 ? ? ? 1/2 2 ? 0 2 ? ? 3 ? 1 ? ? ? 0/1/2 0/1/2 ? ? ? ? 0 2 ? 3 ? 2 ? 0 ? ? ? ? ? 2 1 ? 0 0 0 1 0 1 2 ? ? ? ? ? ? ? ? ? ? ? ? ? ? ? ? ? ? ? ? ? ? ? ? ? ? ? ? ? ? ? ? ? ? ? ? ? ? ? ? ? ? ? ? ? ? ? ? ? ? ? ? ? ? ? ? ? ? ? ? ? ? ? ? ? ? ? ? ? ? ? ? ? ? ? ? ? ? ? ? ? ? ? ? ? ? ? ? ? ? ? ? ? ? ? ? ? ? ? ? ? ? ? ? ? ? ? 0/1 0 1 0 1 ? 2

*Parapontoporia sternbergi*

? 0 2 1 0 1 2 0 2 3 1 0 0 ? ? 0 0 1 1 ? ? 1 ? 8 0 0 ? ? ? ? ? ? ? ? ? ? 8/9 0 1/2 0 1 0 ? ? ? ? 2 ? 3 1 0 1 1 ? ? ? 2 ? 0 ? ? ? 0 0/1 0 1 2 1 1 0 1 1 0 4/5 1 2 5 1 1 7 0 0 0 0 2 ? 0 0 0 0 0 1 0 ? ? ? ? ? ? ? ? ? ? ? ? ? ? ? 0 0 ? 0 0 2 1 0 1 0 1 3 1 5 1 2 2 1 ? 0 0 0 0 0 ? 2 ? 3 0 ? 0 0 0 ? ? 0 1 2 2 0 0 ? 1 2 0 ? 0 2 2 1 0 2 ? ? 3 ? 1 ? 1/2/3 0 0/1/2 0/1/2 0 ? ? ? 0 2 ? 3 ? 2 0 0 3 0 0 ? 0 ? ? 1 0 0 0 2 0 1 2 ? ? ? ? ? ? ? ? ? ? ? ? ? ? ? ? ? ? ? ? ? ? ? ? ? ? ? ? ? ? ? ? ? ? ? ? ? ? ? ? ? ? ? ? ? ? ? ? ? ? ? ? ? ? ? ? ? ? ? ? ? ? ? ? ? ? ? ? ? ? ? ? ? ? ? ? ? ? ? ? ? ? ? ? ? ? ? ? ? ? ? ? ? ? ? ? ? ? ? ? ? ? ? ? ? ? ? 0 0 1 0 ? 1 2

*Brachydelphis mazeasi*

? 0 1 0 0 0 2 0 3 2 2 0 0/1/2 ? 1 0 0 1 0 1 ? 0 ? 5 0 ? ? ? ? ? ? ? ? ? ? ? ? ? ? ? ? ? ? ? ? 0 3 0 0 1 1 1 ? ? 1 ? 0 1 0 ? 0 1 0 1/2 0 1 2 2 1 0 1 2 0 4 1 2 5 0 1 7 1 0 0 0 1 ? 0 0 0 1 0 1 0 ? ? ? ? ? ? ? ? ? ? ? ? ? ? ? ? 0 ? 1 0 1 ? 0 0 0 1 2 0 4 2/3 1 2 0 0 1 1 0 0 0 ? 2/3 0 3 0 ? 0 ? ? 0 0 0 1 0 1 0 0 0 0 2 2 ? 0 ? 1 1 0 2 0 ? 3 1 0 ? 2/3 ? 3 0/1/2 0 ? ? ? 1 ? ? 3 ? 1 0 ? 1/2 0 0 0 2 3 0 1 0 0 0 1 0 0 2 0 2 0 1 0 1 0 0 0/1 ? 1/2 ? 0 1 1 0 0 0 3 1 1 0 1 2 0 0 1 1 0 0 2 1 0 0/3 3 4 0/1 0 1 1 0/1 0 ? 0 0/1 0 0/1 1 1 1 1 1 ? ? ? ? ? 1 ? ? ? ? ? ? ? 0 1 1 2 ? 0 ? 1 1 ? ? ? ? ? ? ? ? ? ? ? ? ? ? ? ? ? ? ? ? 0 ? ? ? 1 1 1 ? ? ? ? ? ? 1 0 1 0 1 0 1

*Orycterocetus crocodilinus*

? 0 1 0 0 0 1 0 3 2 2 0 0 ? 0 0 0 1 0 1 ? 0 0 4 0 ? ? ? ? 0 ? ? 1 ? ? ? ? ? ? ? ? ? ? ? ? 0 1 1 1 1 1 1 1 ? 1 ? 1 0 0 ? 0 0 0 1 0 0 2 0 1 2 0/1 2 0 3/4 1 2 4/5 0 0 5 1 0 0 1 0 1 1 1 0 0 0 0 0 ? ? ? ? ? ? ? ? ? ? ? ? ? 0 4 0 ? ? 1 0/1 2 0 0 0 ? ? 4 ? 2/3 1 ? ? ? ? 2 1 0 0 0 0 2 1 3 0 ? 0 0 ? 1 1 2 0 2 2 0 0 0 0 2 1 ? 0 0 0/1 ? 0 0 ? 2 1 1 0 0 3 ? 0/1/2 0/1/2 ? 1 1 3 1 2 ? 2 ? 2 0 0 2 0 0 0 3 1 1 ? 0 1 2 2/3 1 1 2 ? ? 1 0 0 2 1 0 1 1 2 1/2/3 0 0 1 1 0 0 2 0 2 0 1 2 1 0 1 1 0 1 2 1 0 0 3 1 0 0 0 2 1 0 1 1 2 0 0/1 1 1 0 0 ? ? 0 1 1 1 0 0 0 1 0 1 1 ? 1 1 2 2 1 0 0 1 1 0 2 0 0 ? ? ? ? ? ? ? ? ? ? ? ? ? ? ? ? ? ? ? ? ? ? ? ? ? ? ? ? ? ? ? ? ? ? ? ?

*Kentriodon pernix*

? 0 1 0 0 0 2 0 3 2 1 0 0 ? 1 0 0 1 0 ? ? 0 0 7 0 0 ? ? 0 0 0 0 1 ? ? ? 9 0 1 ? 0 0 1 ? 1 0 1 0 2/3 1 0 1 1 ? 3 1 2 0 0 ? 1 0 0 1 0 1 2 0 1 0 1 1 0 4 1 2 5 0 0 5 0 0 0 0 1 ? 0 0 0 0 0 1 0 ? ? ? ? ? ? ? ? ? ? ? ? ? 0 4 0 1 ? 1 0 1 0 0 0 0/1 4 1 1 4 2 1 2 0 ? 1 1 0 0 0 ? 2 ? 3 0 ? 0 0 0 0 1 0 1 1 2 0 0 0 1 2 0 ? 0 1 0/1 1 0 2 0 ? 3 ? 0 ? 3 0 3 3 ? ? ? ? 0 ? ? 3 ? 2 0 0 2 0 0 0 3 3 1 1 0 0 1 1/2 0 1 2 0 0 1 0 0 2/3 0 0 1 3 2 1 0 1 1 1 0 0 3 1 1 0 0 2 0 0 0 1 0 1 2 1 0 0 3 4 0 0 2 0 0 0 3 1 0 0 1 1 1 0 0 1 1 0 0 1 0 1 0 1 0 0 1 0 0 1 1 0 2 1 1 0 1 1 1 2 0 0 2 0 0 0 6 5 0 1 1 0 2 4/5/6/7 ? ? ? ? ? ? ? ? ? ? ? ? ? ? ? ? ? 0 0 0 0 1 0 2

*Pliopontos littoralis*

? 0 1 0 0 0 2 ? ? 3 0/1 0 0 ? 1 0 0 1 0 1 ? 0 0 6 0 0 ? ? 1 0 0 1 1 ? ? ? 8/9 0 ? 0 ? 0 1 ? ? 0 2/3 0 1/2 1 1 1 1 ? 0/1 ? 0 1 0 ? 1 0 0 1/2 0 1 2 2 1 0 1 2 0 4 1 2 5 0 0 7 1 0 0 0 1/2 ? 0 0 1 0 0 1 0 ? ? ? ? ? ? ? ? ? ? ? ? ? ? ? 2 0 0 0 0 2 0 0/1 0 0 1/2 2 1 5 2/3 1 1/2 0 0 1 1 1 0 0 0 2/3 0 3 0 ? 0 0 ? 0 2 0 1 0/1 1 1 0 0 1 2 1/2 ? 0 1 0/1 1 ? 2 0 ? 3 ? 0 ? 2 0 3 3 0 ? ? ? 1 ? ? 3 ? 2 0 0 2 0 0/1 0 0 3 0 1 0 0 2/3 2 0 ? 2 ? ? 0 1 0 1 0 0 1 ? 2 ? 0 0 ? 1 0 0 3 2 1 0 1 2 0 0 1 1 0 1 1 1 0 0 3 4 1 0 1 0 1 0 ? 1 2 0 1 1 1 1 1 1 0 0 1 ? ? 1 0 1 0 0 0/2 0 ? 1 0 0 2 1 0 1 1 1 1 2 0 0 ? 1 0 0 2/3/4 1/2/3/4/5/6 0 1 1 1 1/2 4/5/6/7 ? ? ? ? ? ? ? ? ? ? ? ? ? ? ? ? ? 0 0 2 0 1 0 1

*Atocetus nasalis*

? 0 1 0 0 0 2 0 0/1 2 1 0 0 ? 1 0 0 2 0 1 ? 0 0 6 0 0 ? 0 1 0 0 1 1 ? ? ? 6 0 1 1 0 0 1 0 1 0 2 0 1 1 0 1 1 ? 0 1 0 0 0 ? 0 1 0 2 0 1 2 0 1 0 1 2 0 2 1 2 5 0 0 5 0 1/2 0 0 1 ? 0 0 0 0 0 1 0 ? ? ? ? ? ? ? ? ? ? ? ? ? 1 4 0 0 0 ? 0 2 1 0 0 1 5 2/3 1 4 2 0 2 1 0 1 0 0 0 0 0 2 ? 3 0 ? 0 ? ? 0 ? 0 0/1 2 2 0 0 ? 0/1 1/2 1 ? 0 ? 2 1 ? 2 0 ? 3 ? 0 ? ? 0 0/1/2 3 0 ? ? ? 0 ? ? 3 ? 1 ? 2 ? 0 0 0 ? 3 0 ? ? ? ? ? ? ? ? 0 2 0 0 0 2 1 0 1 2/3 1 ? 0 1 1 1 0 0 2/3 1 1 ? 0 2 ? 0 1 1 0 1 3 2 0 0 3 4 1 0 1 0 1 0 3/4 0 ? 0 1 1 2 0 0 1 ? ? 0 ? ? 1 ? ? ? ? 1 ? ? 0 1 0 2 1 1 0 1 1 ? 2 ? 0 ? ? ? ? ? ? ? ? 1 1 ? ? ? ? ? ? ? ? ? ? ? ? ? ? ? ? ? ? ? ? 0 0/1 0 1 0 2

*Albireo* *whistleri*

? 0 1 0 ? 0 2 0 0 2 1 0 0 ? 0 0 0 1 0 1 ? 0 ? ? 0 0 0 0 1 0 0 1 1 ? 1 1 7 0 0 1 0 0 1 ? 0 0 2/3 0 3 1 0 1 1 ? 1 1 2 0 0 ? 1 0 0 2 0 1 2 2 1 0 0 1 ? 5 2 2 5 0 1 6 1 0 ? 0 1 ? 0 0 0 1 0 1 0 ? ? ? ? ? ? ? ? ? ? ? ? ? ? ? 0 0 0 1 0 1 1 0 1 0 2 2 0 5 3 2 1 0 ? 1 0 0 0 0 0 1 ? 2 0 ? 0 0 0 0 1 0/1 0 2 2 0 0 0 0 1 1/2 ? 0 2 0/1 1 0/1 ? ? ? 2/3 1 0 0 ? 0 0/1/2 0/1/2 0 ? ? 0/1 0 0 ? 3 ? 1 0 0 2 0 0 ? 1/2/3 3 1 1 0 0 0 2 0 1 2 0 2 0 0 0 2 1 0 1 ? 1 ? 0 1 1 1 0 0 2 1 1 0 1 2 0 0 0 1 0 0 3 1 0 0 3 4 1 0 1 0 1 0 ? 1 0 0 1 2 1 0 0 1 0 0 0 1 0 ? 0 ? 0 0 1 1 1 0 1 0 2 1 1 0 1 1 1 1 ? 0 ? ? ? ? ? ? ? ? ? ? ? ? ? ? ? ? ? ? ? ? ? ? ? ? ? ? ? ? ? ? ? ? ? ? ? ?

*Simocetus rayi*

? 0 1 0 0 0 1 0 4 2 1 0 0 ? 1 0 0 1 0 0 ? 0 4 1 1 1 0 0 1 1 0 1 0 0 0 ? 4 1 0 1 0 1 1 0/1 ? 0 1 0 0 1 0 1 1 ? 0 ? 3 0 0 ? 0 1 0 2 0 1 0 0 1/2 ? 0 2 0 2 2 2 3 ? ? 3 0 0 0 0 1 ? 0 0 0 1/2 0 0 0 ? ? ? ? ? ? ? ? ? ? ? ? ? 0 2 1 0 ? 0 0 1 0 0 0 0 2 1 1 1 0 0 2 0 ? 1 0 0 0 0 0 0 ? 1 1/2 ? 1 0 ? 0 0 1 1 3 1 ? 0 0 2 1 0 0 1 1 0/1 0 0 0 ? 1 2 ? 0 0 0 0 0 0 ? 1 0 3 0 0 ? 2 ? ? 0 0 1 ? ? 0 3 3 0 1 0 0 2 2 0 0 0/1 ? ? 0 0 0 2 0 1 ? 0/1 1 3 0 1 ? 0/1 0 0 ? 1 1 1 0 2 0 ? 1/2/3 0 1 ? ? ? 0 ? ? ? ? ? ? ? ? ? 0/1 ? ? 0 ? ? ? 0 0 1 ? ? ? ? ? 1 ? ? ? ? ? ? 0 0 0 1 ? ? ? ? ? ? ? ? ? ? ? ? ? ? ? ? ? ? ? ? ? ? ? ? ? ? ? ? ? ? ? ? ? ? ? ? ? ? ? ? ? ? ? ? ? ?

*Ninoziphius platyrostris*

? 0 1 0 0 ? ? ? ? 1/2 ? 0 ? ? ? 0 2 ? 0 1 ? 0 0 7 0 ? 0/1 1 ? ? ? ? ? ? 0/1 2 9 0 1 0 0 0 1 0 ? ? ? ? ? ? ? ? ? ? ? 1 ? ? ? ? ? ? 0 ? ? ? 2 0 1/2 ? 1 ? 0 4/5 1/2 2 4/5 ? ? 7 0 ? ? ? ? ? 0 0 ? ? ? ? 0 ? ? ? ? ? ? ? ? ? ? ? ? ? ? ? ? ? 0 ? ? ? ? ? ? ? ? ? ? 4 ? ? ? ? ? ? ? ? ? ? ? ? ? ? ? ? ? ? 0 1 1 0 0 1 2 0 0 0 2 ? ? ? ? ? 0/1 1 0 1 ? 2 3 1 0 0 1 0 0 0 ? 2 1 1 0 1 ? 3 ? 1 0 0 2 0 0 0 3 1 1 1 0 1 0 ? 0 1 2 ? ? 1 2 0 3 0 0 1 ? 2 3 0 1 1 0 0 0 2 1 1 0 0 0 1 0 1 1 0 1 3 2 1 1 ? ? 1 1 3 2 1 0 ? 0 1 0 0 1 2 0 0 1 0 0 0 1 ? 0 0 1 1 0 0/2 ? 0 1 1 2 2 1 0 0 0 0 0 0 0 0 ? 0 1 0 ? ? ? ? ? ? ? ? ? 0 0 0 ? ? ? ? ? ? ? ? ? ? ? ? ? ? ? ? ? ? ? ?

*Meherrinia* *isoni*

? ? ? ? ? 0 2 ? ? 1/2/3 ? 0 0 ? ? 0 0 1 0 1 ? 1 ? 1/2/3/4/5/6/7/8 0 ? ? ? ? ? ? ? ? ? ? ? ? ? ? ? ? ? ? ? ? 0 2/3 0 ? 1 0 1 1 ? 1/2 ? ? ? 0 ? 1 0 0 2 0 1/2 2 2 1 0 1 2 0 3/4 1 2 4/5 0/1 0 6/7 1 0 0 0 1 ? 0 0 1 0 0 1 0 ? ? ? ? ? ? ? ? ? ? ? ? ? ? ? 0 0 0 0/1 0 1 ? 1 0 0 1 2 1 4/5 ? 1 2 0 0 0 1 ? 0 0 ? 2/3 ? 3 0 ? 0/1 ? ? ? ? ? ? ? ? ? ? ? ? 0 ? ? 0 ? 0/1 1 0 2 0 ? 2/3 ? ? ? ? ? 0/1/2 0/1/2 ? ? ? ? ? ? ? ? ? ? ? ? ? ? ? ? ? ? ? ? ? ? ? ? ? ? ? ? ? ? ? ? ? ? ? ? ? ? ? ? ? ? ? ? ? ? ? ? ? ? ? ? ? ? ? ? ? ? ? ? ? ? ? ? ? ? ? ? ? ? ? ? ? ? ? ? ? ? ? ? ? ? ? ? ? ? ? ? ? ? ? ? ? ? ? ? ? ? ? ? ? ? ? ? ? ? ? ? ? ? ? ? ? ? ? ? ? ? ? ? ? ? ? ? ? ? ? ? ? ? ? ? ? ? 0 2 0 1 0 1 1/2

*Stenasodelphis russellae*

? ? ? ? ? ? 2 ? ? ? 3 0 0 ? 1 ? ? ? ? ? ? ? ? ? ? ? ? ? ? ? ? ? ? ? ? ? ? ? ? ? ? ? ? ? ? 0 ? ? 0 1 0 ? 1 ? 1 ? 1 0 0 ? ? ? 0 1 0 1/2 2 ? 2 1 ? 1 0 3/4/5/6 1 2 3/4/5 1 ? 5/6/7/8 1 0 1 0 1 ? 0 0 1 ? 0 1 0 ? ? ? ? ? ? ? ? ? ? ? ? ? ? ? 0 0 0 1 0 ? ? 0 0 1 1 2/3 1 2/3/4/5 ? 0 2 1 0 ? ? ? 0 0 ? ? 0 ? 0 ? 0/1/2 ? ? ? ? ? ? ? ? ? ? ? ? ? ? ? ? ? ? 1 ? 1/2 ? ? 2/3 ? ? ? ? ? 3 ? ? ? ? ? ? ? ? ? ? ? ? ? ? ? ? ? ? ? ? ? ? ? ? ? ? ? ? ? ? ? ? ? ? ? ? ? ? ? ? ? ? ? ? ? ? ? ? ? ? ? ? ? ? ? ? ? ? ? ? ? ? ? ? ? ? ? ? ? ? ? ? ? ? ? ? ? ? ? ? ? ? ? ? ? ? ? ? ? ? ? ? ? ? ? ? ? ? ? ? ? ? ? ? ? ? ? ? ? ? ? ? ? ? ? ? ? ? ? ? ? ? ? ? ? ? ? ? ? ? ? ? ? ? ? 1 1 1 1 0 ? ?

*Auroracetus bakerae*

? ? ? ? 0 ? 1/2 ? ? ? ? 0 0 ? ? ? ? ? ? ? ? ? ? ? ? ? ? ? ? ? ? ? ? ? ? ? ? ? ? ? ? ? ? ? ? 0 ? 0 ? 1 ? ? ? ? ? ? ? ? ? ? 1 1 1 ? ? 0 2 2 1 0 1 1 0 4 1/2 2 4/5 0 0 6 0 0 ? 0 1 ? 0 0 1 ? 0 1 0 ? ? ? ? ? ? ? ? ? ? ? ? ? ? ? 0 0 0 0 0 ? 0 1 0 1 1 4 ? 4/5 ? ? ? ? 0 ? ? 0 0 0 0 ? ? 3 0 ? 0/1/2 ? ? ? ? ? ? ? ? ? ? ? ? ? ? ? ? ? ? 1 ? 1/2 0 ? 2 ? ? ? ? ? ? 3 0 ? ? ? ? ? ? ? ? ? ? ? ? ? ? ? ? ? ? ? ? ? ? ? 0 ? 2 ? ? ? ? ? ? ? ? ? ? ? ? ? ? ? ? ? ? ? ? ? ? ? ? ? ? ? ? ? ? ? ? ? ? ? ? ? ? ? ? ? ? ? ? ? ? ? ? ? ? ? ? ? ? ? ? ? ? ? ? ? ? ? ? ? ? ? ? ? ? ? ? ? ? ? ? ? ? ? ? ? ? ? ? ? ? ? ? ? ? ? ? ? ? ? ? ? ? ? ? ? ? ? ? ? ? ? ? 2 1 1 0 0 1

*Protophocoena minima*

? 0 0 0 0 0 2 0 3 2 1 0 0 ? ? 0 0 ? 0 1 ? 0 ? ? ? ? ? ? ? ? ? ? ? ? ? ? ? ? ? ? ? ? ? ? ? 0 ? 0 ? ? ? ? ? ? ? ? 0/1 0/1 0 ? ? ? 0 ? 0 ? 2 2 1 0 1 1 0 ? ? ? ? ? ? ? 0 0 ? ? 1 ? 0 0 ? 0 0 1 0 ? ? ? ? ? ? ? ? ? ? ? ? ? ? ? ? 0 ? ? 0 1 ? ? 0 0 1 ? 1 ? ? ? 2 ? 0 0 ? ? ? ? ? ? ? ? ? ? ? ? ? ? ? ? ? ? ? ? ? ? ? ? ? ? ? ? 0/1 1 ? 2 0 ? 3 ? ? ? ? ? ? ? ? ? ? ? ? ? ? ? ? ? ? ? ? ? ? ? ? ? ? ? ? ? ? ? ? ? ? ? ? ? ? ? ? ? ? ? ? ? ? ? ? ? ? ? ? ? ? ? ? ? ? ? ? ? ? ? ? ? ? ? ? ? ? ? ? ? ? ? ? ? ? ? ? ? ? ? ? ? ? ? ? ? ? ? ? ? ? ? ? ? ? ? ? ? ? ? ? ? ? ? ? ? ? ? ? ? ? ? ? ? ? ? ? ? ? ? ? ? ? ? ? ? ? ? ? ? ? ? ? ? ? ? ? ? ? 0 1 0 0 ? 1

*Ischyrorhynchus vanbenedeni*

**0** 0 ? 0 0 0 2 0 **0** 3 ? 0 **?** ? ? 0 0 **2** 0 1 ? 0 **0** ? 0 **1** ? ? ? ? ? ? ? ? ? ? ? ? ? ? ? ? ? ? ? 2 **2** 0 2 ? ? ? ? ? ? ? ? ? 0 ? ? ? 0 ? 0 1 2 2 ? ? ? **2** 0 5 ? 2 5 **0** **?** **5** 1 0 ? 0 1 **1** 0 0 **1** **1** 0 1 0 ? ? ? ? ? ? ? ? ? ? ? ? ? 0 ? 0 0 ? ? 0 **1** **0** 0 1 0 2 1 1 4/5 ? 2 0 0 0 0 ? 1 0 0 ? 2 1 3 0 ? 0 ? 1 0 0 0 1 ? ? ? 0 1 0 2 1 0 ? ? 0/1 1 ? 2 0 ? 3 ? ? ? ? ? ? ? ? ? ? ? ? ? ? 2 ? ? ? ? ? ? ? ? ? 1 ? ? ? ? ? ? ? ? ? ? ? ? ? ? ? ? ? ? ? ? ? ? ? ? ? ? ? ? ? ? ? ? ? ? ? ? ? ? ? ? ? ? ? ? ? ? ? ? ? ? ? ? ? ? ? ? ? ? ? ? ? ? ? ? ? ? ? ? ? ? ? ? ? ? ? ? ? ? ? ? ? ? ? ? ? ? ? ? ? ? ? ? ? ? ? ? ? ? ? ? ? ? ? ? ? ? ? ? ? ? ? ? ? ? ? ? **0** **0** 0 0 **0** ? 2

*Papahu taitapu*

? ? ? 0 0 0 2 0 ? 1/2 1 0 0 ? 1 0 0 1 0 1 ? 0 0 3/4/5/6/7/8 0 ? ? ? ? ? ? ? ? ? ? ? ? ? ? ? ? ? 1 ? 0 0 3 0 2 1 1 1 1 1 0 1 0/3 0 0 ? 1 0 0 2 0 1 1 0 2 0 1 1 0 3 1 2 4 0 0 5 0 0 0 0 1 ? 0 0 0 0 0 0 0 ? ? ? ? ? ? ? ? ? ? ? ? ? 1 4 1 0 ? 0 0 1 0 0 0 0 1 1 ? 3 3 1 1 0 0 1 0 0 0 0 0 1/2 1 2 ? ? 1 0 ? 0 1 1 1 1 1 0 0 0 1 1 0/1 0 0 1 0 1 0 1/2 0 ? 2 0 0 ? ? ? 0 0 0 ? ? ? 1 ? ? 2 0 2 ? ? 2 1 2 ? ? 3 ? 1 0 ? ? 2 ? ? ? ? ? 0 1 0 3 0 1 1 ? 2 3 0 1 ? ? 0 0 ? ? 1 0 0 2 0 0 1 ? ? 2 ? ? 0 0 3 4 0 0 0 0 1 ? ? ? ? ? ? ? ? ? ? ? ? ? ? ? ? ? ? ? ? ? ? ? ? ? ? ? ? ? ? ? ? ? ? ? ? ? ? ? 0 ? ? ? ? ? ? ? ? ? ? ? ? ? ? ? ? ? ? ? ? ? ? ? ? ? ? 0 0 0 0 1 0 0

**3. References**

Aguirre-Fernández G, Fordyce RW. 2014. *Papahu taitapu*, gen. et sp. nov., an early Miocene stem odontocete (Cetacea) from New Zealand. *Journal of Vertebrate Paleontology* 34:195-210.

Geisler JH, Godfrey SJ, Lamber O. 2012. A new genus and species of late Miocene inioid (Cetacea, Odontoceti) from the Meherrin River, North Carolina, U.S.A. *Journal of Vertebrate Paleontology* 32:198-211.
